# De novo identification of CD4^+^ T cell epitopes

**DOI:** 10.1038/s41592-024-02255-0

**Published:** 2024-04-24

**Authors:** Paul M. Zdinak, Nishtha Trivedi, Stephanie Grebinoski, Jessica Torrey, Eduardo Zarate Martinez, Salome Martinez, Louise Hicks, Rashi Ranjan, Venkata Krishna Kanth Makani, Mary Melissa Roland, Lyubov Kublo, Sanya Arshad, Mark S. Anderson, Dario A. A. Vignali, Alok V. Joglekar

**Affiliations:** 1 Department of Immunology, University of Pittsburgh School of Medicine, Pittsburgh, PA USA; 2 Center for Systems Immunology, University of Pittsburgh School of Medicine, Pittsburgh, PA USA; 3 Program in Microbiology and Immunology, University of Pittsburgh School of Medicine, Pittsburgh, PA USA; 4 Microbiology and Immunology Diversity Scholars Program, University of Pittsburgh School of Medicine, Pittsburgh, PA USA; 5 Diabetes Center, University of California San Francisco, San Francisco, CA USA; 6 Tumor Microenvironment Center, UPMC Hillman Cancer Center, Pittsburgh, PA USA; 7 Cancer Immunology and Immunotherapy Program, UPMC Hillman Cancer Center, Pittsburgh, PA USA

**Keywords:** Adaptive immunity, Antigen processing and presentation, Autoimmunity

## Abstract

CD4^+^ T cells recognize peptide antigens presented on class II major histocompatibility complex (MHC-II) molecules to carry out their function. The remarkable diversity of T cell receptor sequences and lack of antigen discovery approaches for MHC-II make profiling the specificities of CD4^+^ T cells challenging. We have expanded our platform of signaling and antigen-presenting bifunctional receptors to encode MHC-II molecules presenting covalently linked peptides (SABR-IIs) for CD4^+^ T cell antigen discovery. SABR-IIs can present epitopes to CD4^+^ T cells and induce signaling upon their recognition, allowing a readable output. Furthermore, the SABR-II design is modular in signaling and deployment to T cells and B cells. Here, we demonstrate that SABR-IIs libraries presenting endogenous and non-contiguous epitopes can be used for antigen discovery in the context of type 1 diabetes. SABR-II libraries provide a rapid, flexible, scalable and versatile approach for de novo identification of CD4^+^ T cell ligands from single-cell RNA sequencing data using experimental and computational approaches.

## Abstract (web summary)

SABR-IIs are engineered bifunctional receptors that enable CD4^+^ T cell antigen discovery.

## Main

A hallmark of the adaptive immune system is the ability to raise antigen-specific responses. This is accomplished for αβT cells through the T cell receptor (TCR), which comprises TCRα and TCRβ chains^1^. Specifically, TCRs from CD4^+^ T cells recognize peptide epitopes on MHC-II or human leukocyte antigen (HLA)-II. The estimated size of the mature TCR repertoire is 10^8^–10^10^ unique TCRs in mice and 10^9^–10^12^ unique TCRs in humans^2–4^. Recognition of foreign antigens such as those from SARS-CoV-2 and tumor neoantigens by CD4^+^ T cells leads to their protective function^5,6^. On the other hand, recognition of self-antigens such as insulin in type 1 diabetes (T1D), leads to pathogenic CD4^+^ T cell responses^7,8^. Furthermore, regulatory T cells can bind to self-antigens and prevent autoimmunity^9^. The specificity of CD4^+^ T cells is key to their function, highlighting a need for antigen discovery approaches tailored for MHC-II and HLA-II^10^.

Traditionally, antigen-specific CD4^+^ T cells have been studied using functional assays that measure proliferation, cytokine release or cytotoxicity^11–14^. These assays are sensitive but are limited to investigating tens of peptides simultaneously. Techniques such as barcoded tetramers can efficiently detect antigen-specific T cells but are limited to the interrogation of 100s of specificities simultaneously^15–20^ and are further limited by the instability of multimers and lower affinities of CD4^+^ TCRs^21,22^. Unbiased approaches such as yeast display and combinatorial peptide libraries have been used to identify epitopes de novo, but these methods often identify nonphysiological epitopes (altered peptide ligands or mimotopes), are highly laborious, and in the case of yeast display, rely on soluble TCR generation^23–26^. Cell-based methods are emerging approaches for TCR-directed antigen discovery. These methods preserve physiological TCR–pMHC interactions, can present large and defined epitope libraries and do not require substantial a priori knowledge of antigen specificity^27–32^. The interchangeability between approaches for MHC-I and MHC-II is not trivial. The utility of cell-based, MHC/HLA-II, antigen discovery was demonstrated by Kisielow et al. using pMHC–TCR (MCR-TCR)^28,33,34^, which allowed for the identification of cognate epitopes by iterative screening against libraries encoded through complementary DNA or defined libraries^34^. More recently, TScan-II was deployed for antigen discovery of CD4^+^ T cells but requires separately engineered antigen-presenting cells (APCs)^35^.

With the increasingly widespread use of single-cell RNA sequencing (scRNA-seq) to interrogate T cell responses, it is paramount that T cell antigen discovery methods can be scaled to investigate tens to 100s of TCRs rapidly^36^. Recently, several algorithms for computational antigen discovery have been reported, including grouping of lymphocyte interactions by paratope hotspots (GLIPH/GLIPH2), distance measure on space of TCRs that permits clustering and visualization (tcrdist/tcrdist3) and clonotype neighbor graph analysis (CoNGA)^37–39^. These algorithms identify TCR specificity groups comprising TCRs that share sequence similarity and/or motifs and are therefore predicted to share antigenic specificity. Recently, ‘reverse epitope discovery’ has been explored to leverage large datasets for comparison of TCR amino acid similarity^40^. Ultimately, Rosati et al. were able to identify public, immunodominant CD4^+^ T cell responses across 59 individuals; however, it remains challenging to predict the antigens of private clonotypes in private datasets, highlighting the need for high-throughput methods that synergize both experimental and computational approaches^10^.

Here we showcase a combination of several methodological advances in applying experimental and computational tools for antigen discovery. First, we report a modular cell-based method for antigen discovery using signaling and antigen-presenting bifunctional receptors to encode MHC/HLA-II molecules presenting covalently linked peptides (SABR-IIs) for mouse and human CD4^+^ T cells. Second, we show de novo identification of epitope specificities of TCRs derived from scRNA-seq data in a mouse model of T1D. Finally, we demonstrate that experimental antigen discovery can be amplified post hoc by computational approaches. Together, we have developed an experimental and computational workflow to rapidly de-convolute the specificity of scRNA-seq-derived CD4^+^ T cells de novo.

## Results

### Signaling and antigen-presenting bifunctional receptors II

**Fig. 1:**
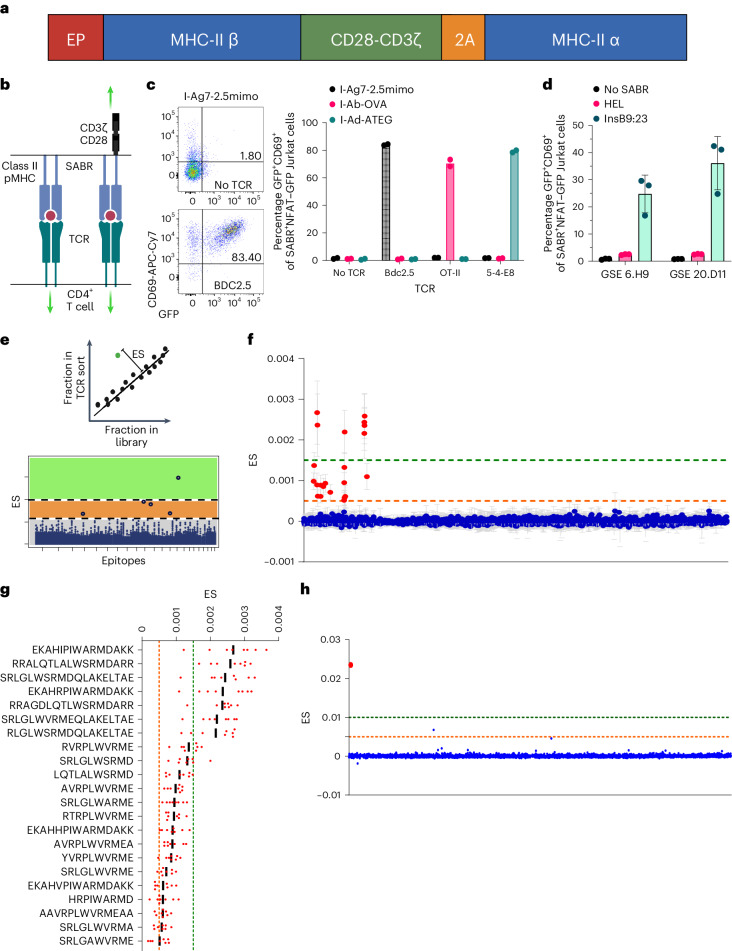
SABR-IIs identify cognate TCR–pMHC interactions for antigen discovery. **a**, A schematic of SABR-II constructs. **b**, Signaling directionality between pMHC:TCR (left) and a SABR-II:TCR (right) **c**, Representative and summary plots for GFP and CD69 expression from SABR-II-expressing NFAT–GFP Jurkat cells after culture with TCR-expressing Jurkat cells. The bar graph indicates the mean of two technical replicates (dots). **d**, SABR co-incubation of Jurkat cells expressing either the GSE.6.H9 or GSE.20.D11 TCR against NFAT–GFP Jurkat cells expressing InsB9:23 or hen egg lysozyme (HEL) in HLA-DQ8 SABR-IIs. Mean and s.d. are plotted from three biological replicates. **e**, Schematic (top) of the ES metric used for putative hit-calling in SABR-II screens. Cartoon ES plot (bottom) of a SABR screen where putative hits (dots/circles) will fall in high- (green) and low-confidence (orange) zones based on positive control TCR–pMHC interactions. **f**,**g**, ES plots from I-Ag7 SABR-II library screens of the BDC2.5 TCR from eight biological replicates. The green and orange lines indicate the high- and low-confidence ES zones, respectively. In **f**, each dot represents the mean for each epitope with s.d. In **g**, the bar represents the mean with each biological replicate plotted as a point for the top 22 putative hits (*x* axis). **h**, ES plot for screen of the GSE.20.D11 TCR against both HLA-A2.1 and HLA-DQ8 SABR libraries simultaneously. Reads were mapped to the DQ8 library and ES was calculated for these epitopes. The InsB9:23 epitope is highlighted by the larger red dot. Source data

**Extended Data Fig. 1:**
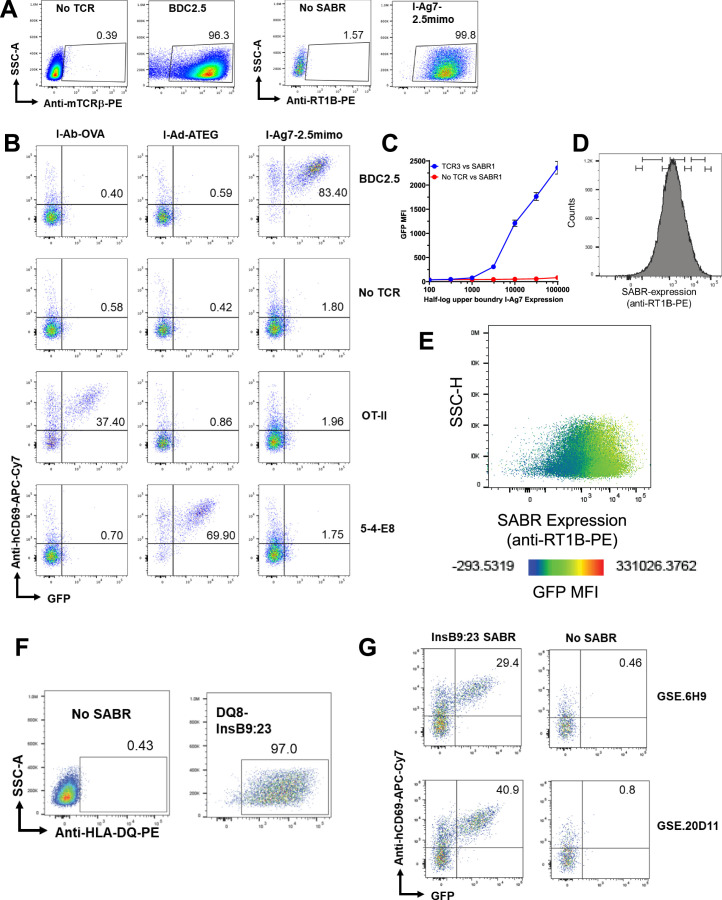
SABR expression and signaling. A. Representative expression of murine TCRβ (Bdc2.5 TCR) and I-Ag7-2.5mimo SABR-II after transduction of Jurkat and NFAT-GFP-Jurkat cells respectively. B. Representative flow cytometry plots of SABR-II expressing NFAT-GFP-Jurkat cells after co-incubation with TCR-expressing Jurkats. The TCRs and SABRs are indicated by rows and column names respectively. C. TCR3 expressing or mock Jurkats were co-incubated against NFAT-GFP-Jurkats cells expressing the I-Ag7 SABR presenting the QVEQLELNAARDPN HIP (SABR1). The GFP MFI was plotted (y-axis) as dots with s.d. (error bars) from technical duplicates against the I-Ag7 levels binned by half logs of the I-Ag7 labeling MFI (x-axis, bins depicted in D). D. Gating strategy to generate the half log bins of I-Ag7 expression in panel C E. Representative pseudo color dot plot depicting the I-Ag7 levels (x-axis) of SABR1 NFAT-GFP-Jurkats co-incubated against TCR3 expressing Jurkats depicted in panel A. The pseudo coloring depicts the GFP MFI for each given event. F. Representative expression of the HLA-DQ8 SABR-II after transduction of NFAT-GFP-Jurkat cells. G. Representative flow cytometry plots of SABR-II expressing NFAT-GFP Jurkat cells after co-incubation with TCR-expressing Jurkats. The respective TCRs and SABRs are indicated by rows and columns respectively. Source data

We have previously described SABRs, which are chimeric receptors containing an extracellular pMHC complex attached to an intracellular CD28-CD3ζ signaling domain. We demonstrated that SABRs can read out TCR–pMHC interactions, allowing the construction of SABR libraries for antigen discovery for class I HLA alleles^27^. We sought to expand this platform to allow antigen discovery for MHC/HLA-II with seamless integration with class I alleles. Here, we created SABRs to present epitopes in MHC-II alleles, by covalently linking the epitope to the β-chain of MHC-II that is attached to the CD28-CD3ζ signaling domains downstream, along with a 2A peptide-linked MHC-II α-chain (Fig. 1a,b). To test whether SABR-IIs could present epitopes to TCRs and induce a signal, we expressed them using lentiviral vectors in NFAT–GFP Jurkat cells, which express green fluorescent protein (GFP) upon NFAT activation and translocation downstream of CD3ζ activation (a kind gift from Y. Chen and A. Weiss). We constructed murine SABR-IIs presenting epitopes in I-Ab, I-Ad and I-Ag7 (Ova, ISQAVHAAHAEINEAGR^41^; ATEG, ATEGRVRVNSAYQDK^42^; and 2.5mimo, YVRPLWVRME^43^, respectively). We co-incubated the SABR-II-expressing NFAT–GFP Jurkat cells with a separate population of Jurkat cells expressing either the BDC2.5 TCR (recognizes I-Ag7-2.5mimo), OT-II TCR (recognizes I-Ab-Ova), 5-4-E8 TCR (recognizes I-Ad-ATEG) or no TCR. Robust GFP and CD69 expression in SABR-II-expressing NFAT–GFP Jurkat cells was observed 18–20 h later in only the correctly paired assays (Fig. 1c and Extended Data Fig. 1a,b). The signal from the NFAT–GFP reporter offered minimal background in absence of a cognate TCR and correlated with surface SABR expression in the presence of a cognate TCR (Extended Data Fig. 1c–e). To demonstrate the application of SABR-IIs for human antigen discovery, we generated SABR-IIs to present the InsB9:23 epitope (SHLVEALYLVCGERG) in HLA-DQ8 (DQA1*0301:DQB1*0302, an HLA-II allele that is associated with increased risk of T1D and celiac disease^44,45^). We confirmed the ability of the DQ8-InsB9:23 SABR-II to present the epitope to two previously described, T1D patient-derived TCRs GSE.6H9 and GSE.20D11 (ref. ^46^). As expected, a high frequency of GFP^+^CD69^+^ cells were found only when the TCRs interacted with the InsB9:23 epitope and not a control hen egg lysozyme epitope (Fig. 1d and Extended Data Fig. 1f,g).

**Extended Data Fig. 2:**
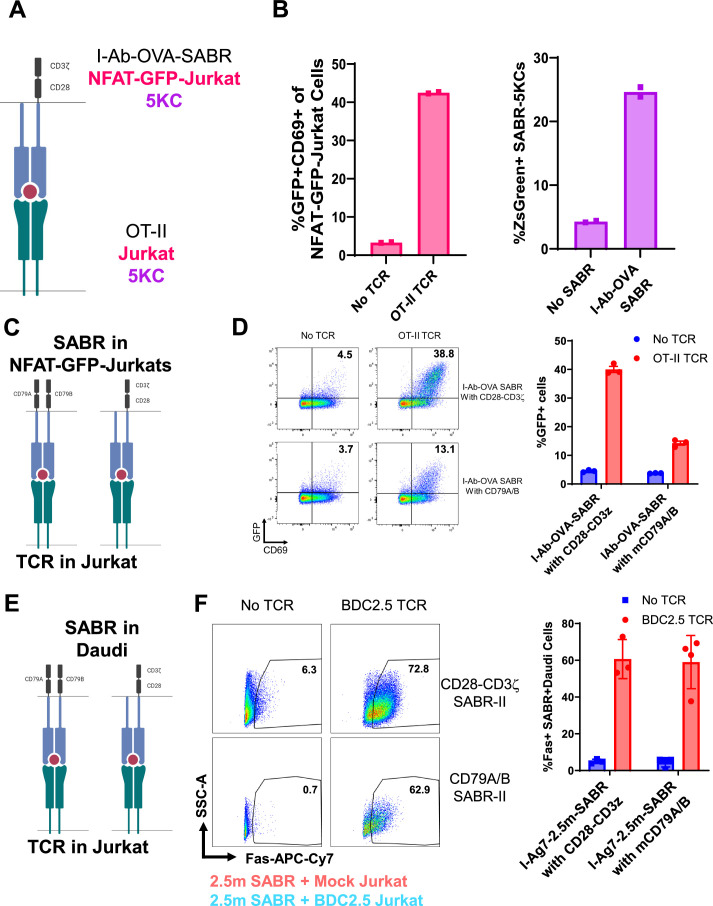
Modularity of SABR-IIs. A. Schematic (color coded by cell type) for corresponding co-incubation assays demonstrating SABR-IIs in both human (Jurkat) and murine (5KC) cell lines. B. OT-II TCR-expressing Jurkat cells against NFAT-GFP-Jurkats expressing the I-Ab-OVA SABR (left) and OT-II expressing 5KC cells against 5KC cells expressing the I-Ab-OVA SABR (right). Bars represent means from two biological replicates (dots). C. Schematic for corresponding co-incubation assays in NFAT-GFP-Jurkat cells demonstrating SABR-IIs with CD28-CD3z or CD79A and CD79B signaling domains. D. Representative flow plots (left) of OT-II TCR Jurkats co-incubated against NFAT-GFP-Jurkats expressing SABR-IIs with either CD28-CD3z(top) or CD79A and CD79B signaling domains. GFP expression was quantified after 24 hr co-incubation (right) with bars depicting the mean and s.d. (error bars) from 3 biological replicates (dots). E. C. Schematic for corresponding co-incubation assays in Daudi Cells demonstrating SABR-IIs with CD28-CD3z or CD79A and CD79B signaling domains. F. Representative dot plots (left) of Bdc2.5 TCR Jurkats co-incubated against Daudi cells expressing SABR-IIs with either CD28-CD3ζ or CD79A and CD79B signaling domains. The percentage of SABR-II+ Daudi cells expressing FAS was quantified by flow cytometry after 72 hr with bars depicting the mean and s.d. (error bars) from 3 biological replicates (dots). Source data

To test the compatibility between human and mouse cells for the function of SABR-IIs, we performed co-incubation assays using 5KC cells (a mouse thymoma line, which was a kind gift from M. Nakayama). We observed that SABR-II–TCR interactions were retained irrespective of the host species (Extended Data Fig. 2a,b). Furthermore, we demonstrated that SABR-IIs consisting of B cell signaling domains (CD79A and CD79B), could also signal through NFAT (Extended Data Fig. 2c,d). As a further demonstration of the modularity of the SABR-II design and its potential for deployment in professional APCs, we expressed SABR-IIs containing either the CD28-CD3ζ or CD79A/B domains in Daudi B cells. We observed that the cognate interaction of both the SABRs with their TCRs resulted in upregulation of surface FAS on Daudi cells, showing that the SABR-II platform can signal in professional APCs^47–49^ (Extended Data Fig. 2e,f).

**Extended Data Fig. 3:**
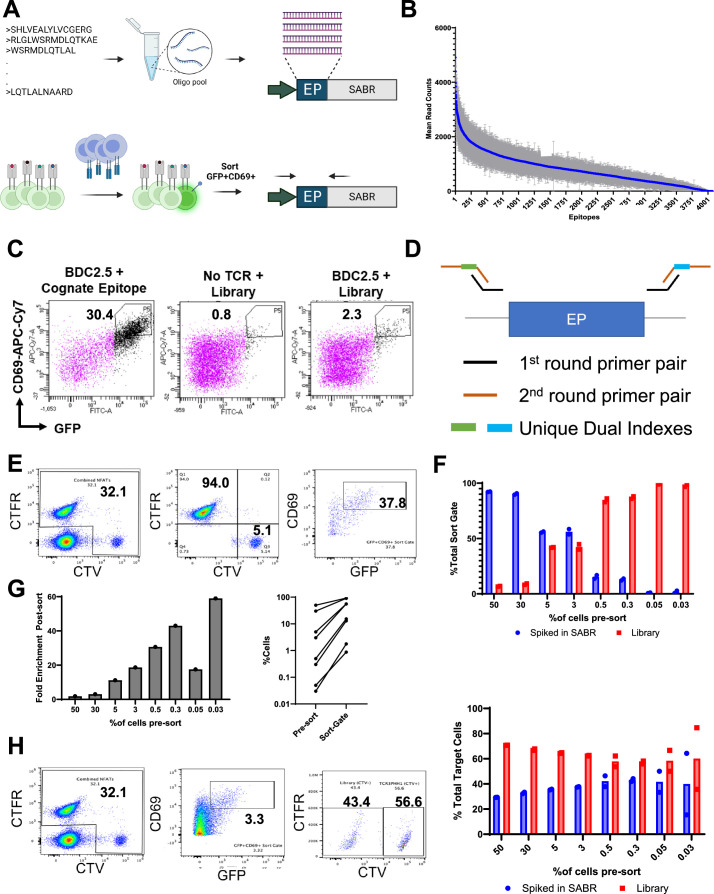
SABR-II library screening. A. Schematics of the SABR-II library cloning and PCR strategy used for targeted reamplification of gDNA from sorted SABR-II library cells. B. The average read count in the libraries across 8 independent experiments is shown (blue dots) with s.d. (error bars). The x-axis denotes the epitope number (ordered in a descending order of mean read counts). C. Representative flow cytometry plots for SABR-II screen sorts. NFAT-GFP-Jurkat cells expressing the SABR-II library were labeled with cell trace violet, gated, and subsequently used to select top 1–2% of GFP/CD69 double positive cells for sorting. D. PCR indexing strategy for epitopes from gDNA for sequencing of SABR-II screens. E-H. NFAT-GFP-Jurkat cells expressing the I-Ag7 library, which contains no TCR3 target epitopes, were labeled with cell trace far red (CTFR+), and NFAT-GFP-Jurkat cells expressing the TCR3 targeted single SABR-II (QVEQLELNAARDPN) were labeled with cell trace violet (CTV+). The two cell types were mixed at decreasing ratios of the TCR3 targeted SABR-II and incubated against TCR3 expressing Jurkats at a 1:1 Jurkat to NFAT-GFP-Jurkat ratio. E. Gating strategy to identify sensitivity and enrichment of target cells in SABR-II library sort gate. Cells were partitioned by cell trace label then separately analyzed for proportion that fall into the sort gate (1–2% CD69+GFP+ NFAT-GFP-Jurkat cells) in the far-right panel. F. Relative proportion of TCR3 targeted SABR-II cells and library cells in the assay that are captured in the sort gate where bars indicate mean from 3 technical replicates. The x-axis indicates the level of spike in of the TCR3 target single SABR-II. G. Left panel shows the fold enrichment (mean of 3 technical replicates) of spiked in TCR3 target single SABR-II cells after sorting. The x-axis indicates the proportion of target cells spike in as in panel F. The right panel shows the pre- and post-sort proportion of spiked in TCR3 target single SABR-II cells. H. Gating strategy to specifically identify specificity of sort gate. Cells are partitioned by cell trace labeling after their appearance in the sort gate. Right panel shows quantification of the proportion of the TCR3 targeted single SABR-II cells that makeup the total sorted cells vs the untargeted library where bars indicated the mean percentage of total sorted cells from two technical duplicates (dots) across the % of target cells spiked in pre-sort (x-axis). Source data

**Extended Data Fig. 4:**
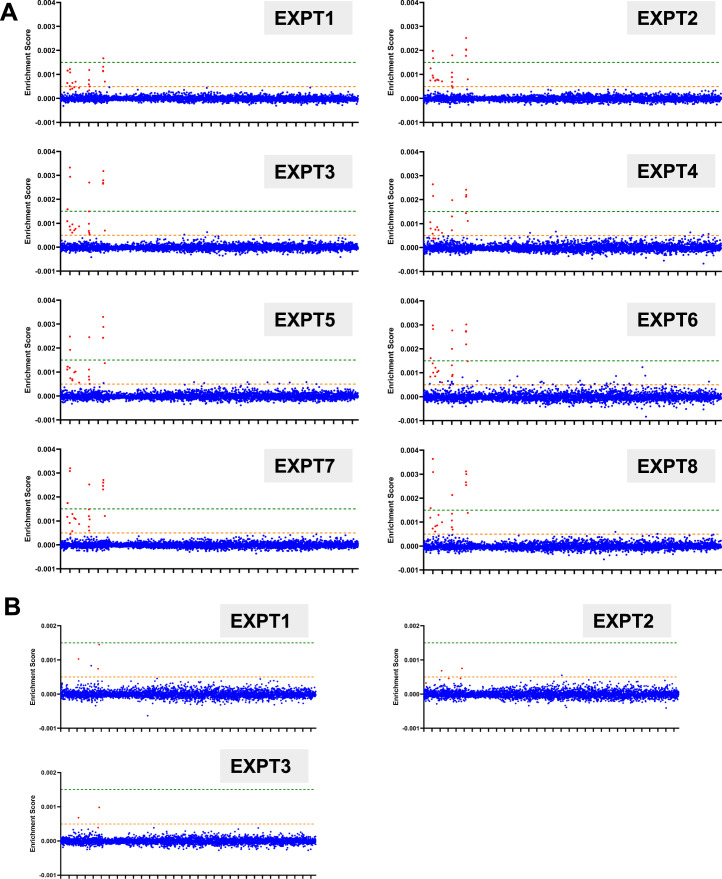
Enrichment score plots from I-Ag7 Library Validations. A. The enrichment score plots for each of 8-independent replicate screen of the BDC2.5 TCR against the I-Ag7-SABR-II library. The resulting high and low confidence thresholds are denoted by green and orange lines respectively. B. The enrichment score for each of 3-independet replicate screen of the 4–8Ins TCR against the SABR-II library. The same high and low confidence thresholds as maintained from experiments in panel A. For every plot each dot represents a single epitope spread across the x-axis and red dots indicate putative cognate epitopes selected for validation. Source data

We then asked whether SABR-IIs could be used to present a library of epitopes for CD4^+^ T cell antigen discovery. To that end, we constructed a SABR-II library to present epitopes derived from pancreatic islets in I-Ag7 by curating a list of 4,075 published epitopes from the Immune Epitope Database (iedb.org)^50^ and a study by Wan et al.^51^ (Supplementary Table 1). Of note, this defined library consisted of unmodified epitopes from endogenous proteins, synthetic mimotopes, deamidated epitopes and hybrid insulin peptides (HIPs) that arise from post-translational fusion and are not genetically encoded in vivo^52,53^. The epitope library was inserted into the I-Ag7 SABR-II backbone through pooled oligonucleotide synthesis, amplification and ligation-free cloning (Extended Data Fig. 3a). The I-Ag7 SABR-II-library was then expressed in NFAT–GFP Jurkat cells. We confirmed that after sequencing, the library accounted for a mean of 708 reads per epitope (Extended Data Fig. 3b). As a proof of concept, we performed co-incubation assays with Jurkat cells expressing the BDC2.5 TCR and sorted the top 1–2% of GFP^+^CD69^+^ cells at a rate of ~20 min per replicate with three replicates per TCR. We extracted the genomic DNA from sorted cells, amplified the SABR portion of the integrated proviruses and subjected the amplicons to Illumina sequencing (Extended Data Fig. 3c,d). The 1–2% sort gate represents >50-fold enrichment of cognate epitopes with minimal loss of signal (Extended Data Fig. 3e–h). Sequence reads were aligned to the I-Ag7 SABR-II backbone and the corresponding epitopes were scored based on their read counts. For each TCR under investigation, an enrichment score (ES) was determined for all the epitopes in a library. In each experiment, three replicates of a sort with TCR-expressing Jurkat cells were performed and reads were counted post-sequencing. In addition, three replicates of the unsorted library were also sequenced. A linear regression model was built using the unsorted library counts and used to determine the expected abundance of each epitope in the library. The ES was calculated based on the difference between the measured and the expected abundance of each epitope on a per-TCR basis (Fig. 1e). Based on ES values, two quantitative thresholds were used to determine putative cognate epitopes of a given TCR. A high-confidence zone containing clear outliers with a high ES and a low-confidence zone containing weak outliers with a moderately high ES were determined (Fig. 1e). This two-tiered strategy was used to call putative hits from screens. All the top-scoring epitopes for the BDC2.5 TCR were known BDC2.5 ligands containing the WXRM(D/E) motif (Fig. 1f,g, enriched ligands in red), a well-characterized trait of the BDC2.5 TCR^43,54–56^. Across several independent experiments there was limited variation in ES values for the same TCRs and several epitopes fell into high- or low-confidence zones consistently (Extended Data Fig. 4a). Using a different TCR that was isolated from NOD mice, 4-8Ins^57^, which recognized the InsB9:23 epitope (SHLVEALYLVCGERG), we observed a similar pattern of ES for cognate epitopes (Extended Data Fig. 4b).

To test whether HLA-DQ8 SABR-II could be used for antigen discovery, we curated a list of insulin B, insulin C and HIP epitopes published by Wiles et al.^58^ and cloned them into the DQ8 SABR-II backbone using the same pooled cloning strategy as the I-Ag7 SABR-II library (Supplementary Table 2). Furthermore, we combined SABR-I (the HLA-A*0201 library reported in our previous work^27^) and SABR-II libraries at a cellular level and screened against the GSE.20D11 TCR. As expected, the cognate epitope of the GSE.20D11 TCR, SHLVEALYLVCGERG (red), was enriched at a high confidence level from a combined class I and II library (Fig. 1h). This demonstrates that a combined library approach using the SABR platform can be implemented to increase throughput. Together, these results demonstrate the ability of SABR-IIs to successfully read out pMHC-II–TCR interactions across species and cell types and serve as a method for CD4^+^ TCR antigen discovery.

### Single-cell profiling of islet-infiltrating CD4^+^ T cells

**Fig. 2:**
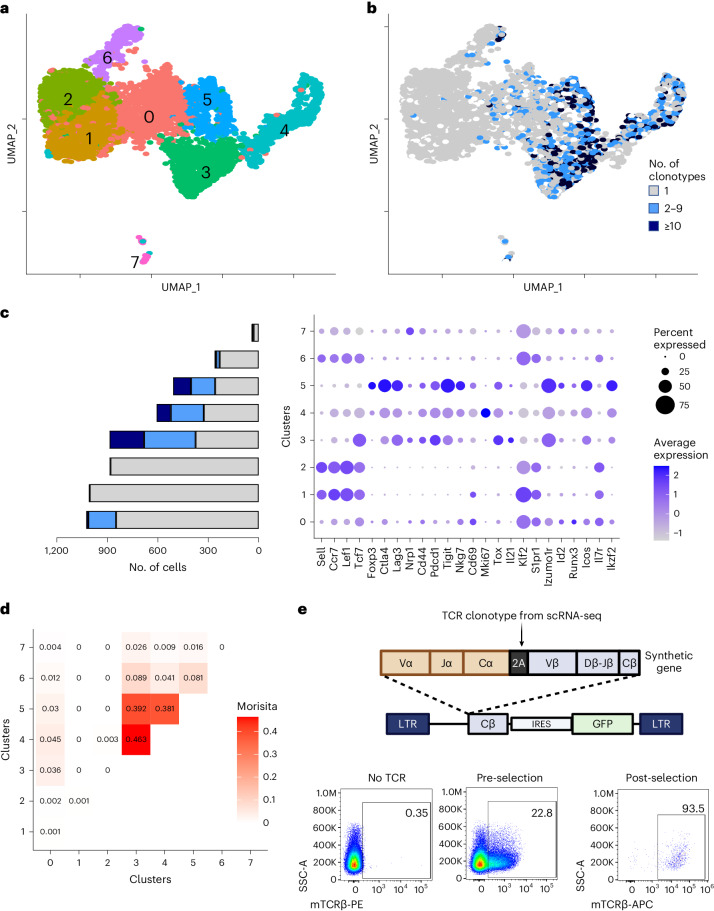
Single-cell RNA sequencing of islet-infiltrating CD4^+^ T cells. **a**, Uniform Manifold Approximation and Projection (UMAP) representations of islet-infiltrating CD4^+^ T cells from 6-, 8- and 10-week-old NOD mice. Hierarchical clusters generated by Seurat are shown in different colors and numbered. **b**, Overlay of clonal expansion on the gene expression cluster UMAP plot. Gray dots represent cells with unique clonotypes, light blue dots represent low (2–9 clonotypes) expansion, dark blue dots represent high (≥10 clonotypes) expansion. **c**. Dot plot of expression of select T cell markers by cluster. Left bar graph depicts cell number on *x* axis with colors to denote clone size from **b** and differential gene expression of select genes across clusters. **d**, Morisita–Horn index plot comparing all TCR sequences across each cluster. **e**, Schematic (top) of TCR cloning strategy into pMIG-II backbone along with representative flow cytometry plots (bottom) of murine TCR levels before and after enrichment in Jurkat cells. Source data

**Extended Data Fig. 5:**
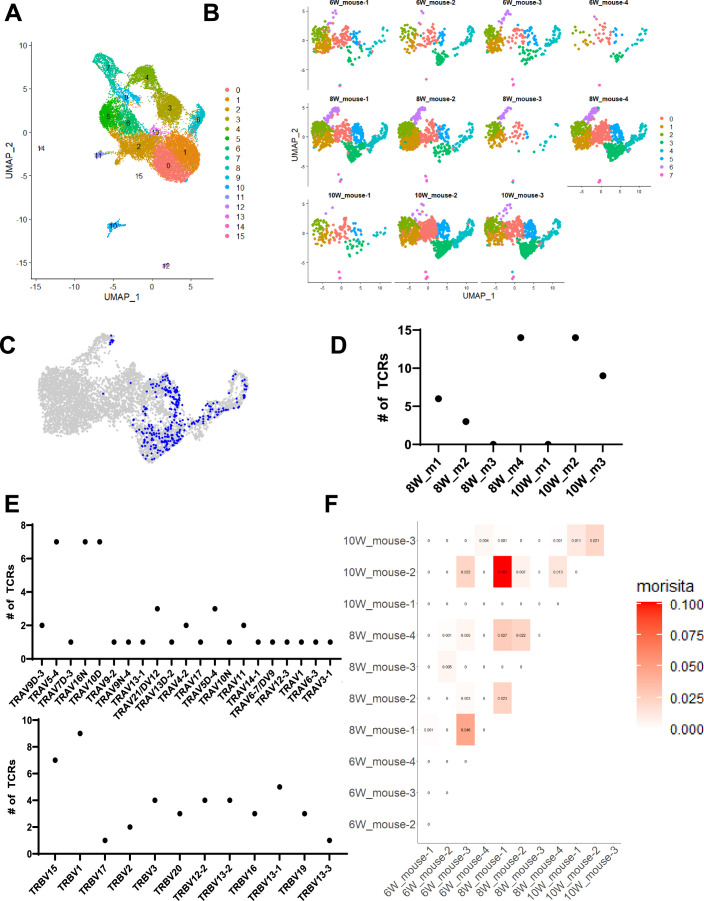
Single-cell RNA-Sequencing of islet-infiltrating CD4+ T cells from NOD mice. A. Hierarchical clustering of total T cells across 11 mice from 6-, 8-, and 10-week-old time points. B. Hierarchical clustering of CD4+ T cells from individual mice across 6-, 8-, and 10-week time points. C. Projection of top 40 expanded CD4+ T cell clones from 8-, and 10-week-old NOD mice onto Seurat clusters using scRepertoire. D. Distribution of top 40 expanded CD4+ TCR sequences across all mice. E. TRAV (top) and TRBV (bottom) usage from top 40 expanded CD4+ TCR sequences across all mice. F. Morisita-Horn index comparing expanded TCR clones across each mouse individually. Source data

We sought to apply SABR-II libraries to identify the specificities of islet-infiltrating CD4^+^ T cells in NOD mice. Although NOD mice recapitulate many features of T1D and share several autoantigens with individuals with T1D^53,59–61^, the overall antigenic landscape of islet-infiltrating CD4^+^ T cells in NOD mice remains undefined. Therefore, we performed scRNA-seq with V(D)J enrichment on T cells from individual pancreatic islets of 6-, 8- and 10-week-old NOD mice. We sorted Thy1.2^+^TCRβ^+^ T cells from 3–4 mice at each time point, combined them using TotalSeq cell-hashing oligonucleotides and proceeded to scRNA-seq using the 10x Genomics platform. In total, T cells from 11 mice were sequenced in three batches and the data were pooled for analysis. Hierarchical clustering in Seurat^62^, followed by bioinformatic gating on CD4^+^ T cells and re-clustering, revealed seven distinct CD4^+^ T cell clusters with no obvious bias between mice (Fig. 2a and Extended Data Fig. 5a,b). Next, we integrated TCR clonotypes with the transcriptomes using scRepertoire^63^ and identified the clonally expanded populations of CD4^+^ T cells (Fig. 2b). Clonal expansion was categorized as single (one clone per TCR), low (2–9 clones per TCR) or medium (≥10 clones per TCR). Clonal expansion was evident in clusters 0 and 3–6 (Fig. 2c). Generally, clonal expansion correlated with the expression of activation and exhaustion markers (*Nkg7*, *Ccl5*, *Lag3* and *Tigit*), whereas naive T cell markers (*Sell* and *Ccr7*) coincided with un-expanded populations. We reasoned that clonally expanded cells within the islets were the most likely to target islet antigens and contribute to β-cell destruction. Therefore, we used clonal expansion as the sole criterion for selecting TCRs for antigen discovery. Overall, clonally expanded TCRs showed a slight skew toward certain Vα and Vβ alleles (Extended Data Fig. 5c–e), as has been reported previously^64,65^. Notably, expanded clones did not segregate solely based on their gene expression as indicated by the high degree of clonal sharing between CD4^+^ TCR clusters determined by the Morisita–Horn Index (Fig. 2d and Extended Data Fig. 5f). Clonally expanded TCRs showed increased expression of *Lag3*, similar to a restrained CD8^+^ T cell phenotype that was reported previously in NOD mice^66^. Further investigations into the transcriptional signatures of expanded T cells were reported previously^67^. Specifically, we identified 35 clonally expanded TCRs for screening, corresponding to 19 TCRs from three 8-week-old mice and 16 TCR from two 10-week-old mice (Supplementary Table 3). We reconstructed the TCRs using a home-brewed Python script that reconstructs full TCRα/β chains using the IMGT TCR allele dataset (Methods)^68^. The reconstructed TCR genes were synthesized through commercial vendors and subcloned into the pMIG-II–IRES–GFP vector containing a partial Cβ-chain derived from the BDC2.5 TCR. TCRs in the pMIG-II vector were then packaged intro retroviruses and expressed in Jurkat cells. Surface expression was confirmed by staining for murine TCRβ followed by flow cytometry. For TCRs with low transduction levels, we enriched the TCRβ^+^ population using either fluorescence-activated cell sorting or magnetic selection and proceeded with antigen discovery with SABR-II libraries (Fig. 2e).

### Identifying cognate epitopes of CD4^+^ TCRs de novo

**Fig. 3:**
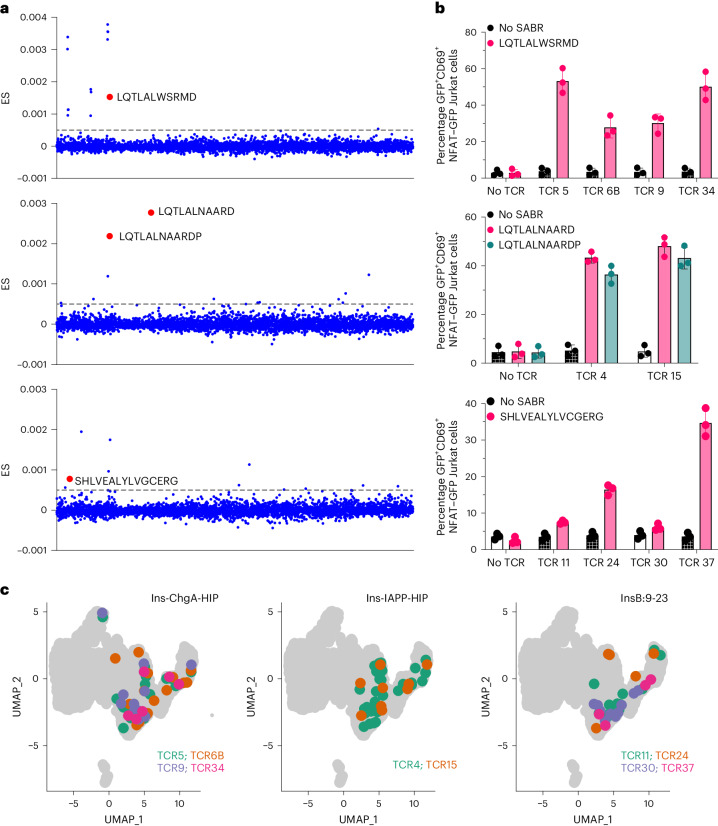
De novo identification of cognate epitopes for expanded CD4^+^ T cells. **a**, Representative SABR-II screen results for three TCRs are shown. Putative non-APL hits are indicated with the epitope sequences and larger red dots. Each dot represents an epitope in the library. The dotted lines indicate threshold for calling putative hits. **b**, Single SABR-II co-incubation assays with TCR-expressing Jurkat cells against NFAT–GFP Jurkat cells expressing SABR-IIs presenting a single epitope (as indicated). GFP^+^CD69^+^ cells in co-incubation assays at 18 h are quantified. Bars show mean and s.d. from three biological replicates. **c**, Projection of antigen-specific CD4^+^ T cell clones onto the UMAP plots of islet-infiltrating CD4^+^ T cells. TCRs are color coded and their cognate epitopes are indicated in the plot title. Source data

**Extended Data Fig. 6:**
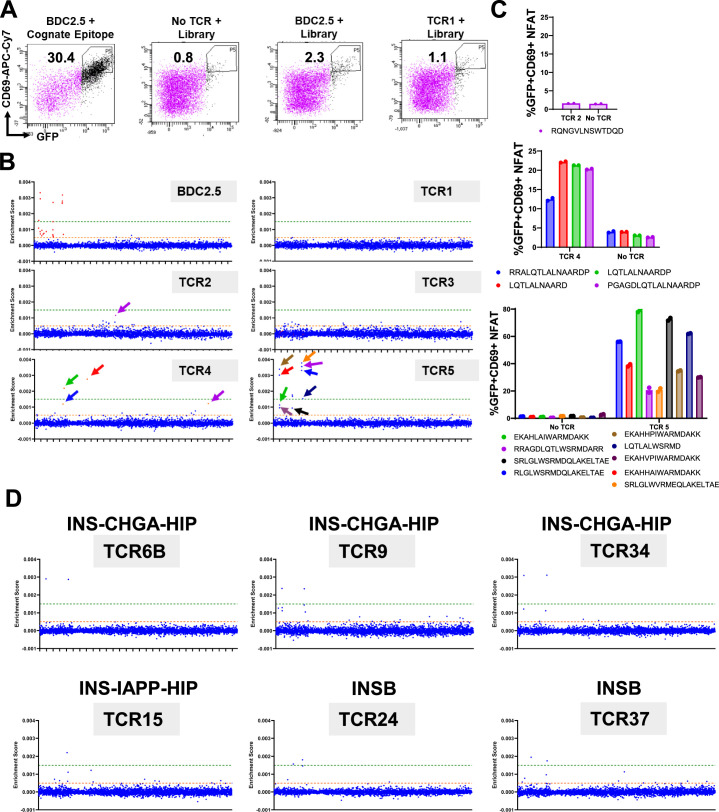
Representative SABR-II screens and hit validation. A. Representative flow sort gating for cell trace violet labeled NFAT-GFP-Jurkat cells expressing the I-Ag7-SABR-II library after co-incubation with Jurkat cells expressing TCRs. Top 1–2% of cells expressing GFP and CD69 were sorted as is shown in the two rightmost panels (gate is constant across panels). B. ES plots from a single sort of 6-TCRs individually. High and low confidence thresholds are denoted by green and orange lines respectively. The colored arrows indicate putative hits tested for validation with single SABR-II assays depicted in panel C. C. Single SABR-II co-incubations for validation of putative hits from the screens in panel B where single SABR-II expressing NFAT-GFP-Jurkat cells were co-incubated against Jurkats expressing the TCR of interest and assayed 18–20 hr later by flow cytometry. Bars indicate means from 2 technical replicates (dots). Top panel depicts non-validated epitope, middle and bottom depict validated synthetic altered peptide ligands (APLs) and physiological epitopes. D. ES plots for TCRs screened against the I-Ag7-SABR-II library that yielded high-confidence putative hits grouped by the highest non-APL epitope. The same high and low confidence thresholds are used from plots generated in Extended Data Fig. 4. Source data

**Extended Data Fig. 7:**
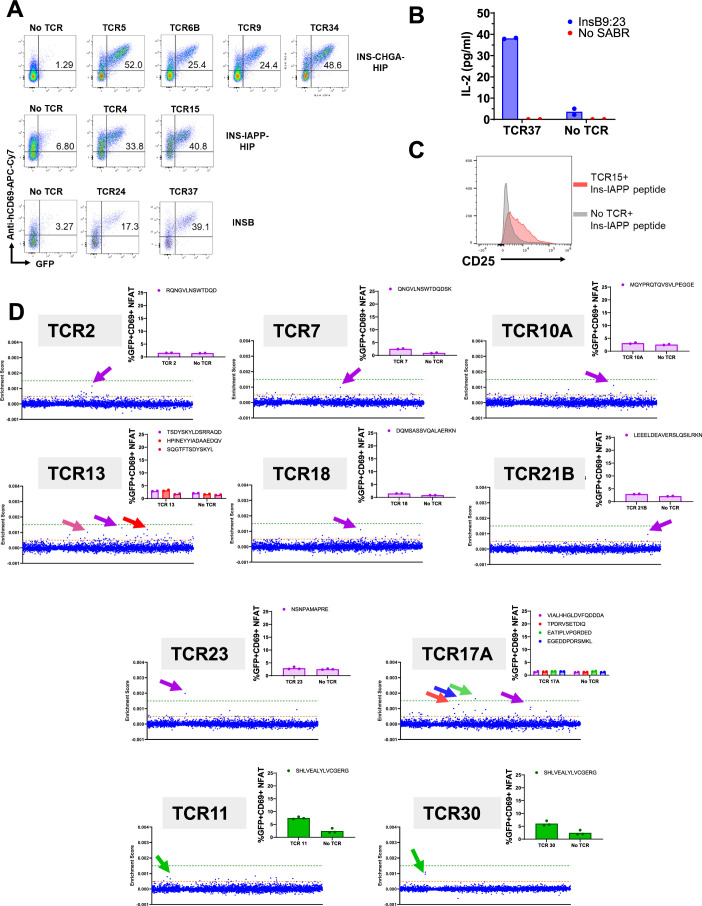
Putative hit validation for high and low confidence hits. A. Representative flow plots from the single SABR-II validations of the putative hits for high-confidence, non-APL hits. B. Murine IL-2 ELISA from 24 hr co-incubation of 5KC cells expressing TCR37 with NFAT-GFP-Jurkat cells expressing the InsB9:23 epitope where bars represent mean of IL-2 sectretion into supernatant from two technical duplicates (dots). C. CD25 expression measured on primary murine CD4+ T cells expressing TCR15 after 24 hr co-incubation with Bone Marrow Dendritic Cells pulsed with either 1μg InsC-IAPP peptide (LQTLALNAARDP) or no peptide. D. ES plots with arrows denoting epitopes tested in the corresponding single SABR-II co-incubations. The same high and low confidence thresholds are taken from Extended Data Fig. 4. The inset plots show single SABR-II validation assays where bars indicate the mean from 2–3 technical replicates (dots). The arrow colors match the epitopes within each inset plot. Source data

We performed systematic screening of the cloned TCRs against the I-Ag7 SABR-II library. Several TCRs along with a positive control (such as BDC2.5 or 4-8Ins) were screened individually against the library for each sort (Extended Data Fig. 6a and Supplementary Table 4). High- and low-confidence ES zones for each screened TCR were defined by the ES values of the control TCR’s cognate epitopes. For all putative cognate epitopes, single SABR-IIs were constructed, expressed in NFAT–GFP Jurkat cells and used for co-incubation with the corresponding TCRs. Co-incubation assays that yielded a GFP signal higher than that obtained in assays with no TCR were determined to be positive and the epitopes deemed true cognate ligands (Extended Data Fig. 6b,c). Using this strategy, we obtained epitopes in the high-confidence zone for eight TCRs (Fig. 3a and Extended Data Fig. 6b,d). Among numerous altered peptide ligands (APLs), these TCRs recognized the physiological InsC-ChgA HIP (LQTLALWSRMD and analogs, recognized by TCR5, TCR6B, TCR9 and TCR34), InsC-Iapp HIP (LQTLALNAARDP and analogs, recognized by TCR4 and TCR15) and InsB9:23 (SHLVEALYLVCGERG and analogs recognized by TCR24 and TCR37). These cognate high-confidence hits were validated using single SABR-II co-incubation assays (Fig. 3b and Extended Data Fig. 7a). Further validations using in vitro mouse interleukin-2 (mIL-2) secretion by TCR-expressing 5KC reporter cells^13^ or CD25 expression by TCR-expressing splenic CD4^+^ T cells upon stimulation with the cognate epitope were performed (Extended Data Fig. 7b,c). Furthermore, low-confidence hits were called for ten TCRs and tested in co-incubation assays. Upon co-incubations, two out of the ten TCRs (TCR11 and TCR30) showed confirmation of reactivity, both recognizing InsB9:23 (SHLVEALYLVCGERG and analogs; Extended Data Fig. 7d and Fig. 3b). Notably, visualization of the cells corresponding to each de-convoluted TCR clone did not reveal overt differences in the transcriptional phenotype of cells recognizing the three different antigens (Fig. 3c). Taken together, these results indicate that SABR-II libraries can successfully identify cognate epitopes of CD4^+^ TCRs among thousands of epitopes for TCR-directed antigen discovery, starting simply from a TCR sequence with little a priori knowledge.

### TCR similarity predictions amplify antigen discovery

**Fig. 4:**
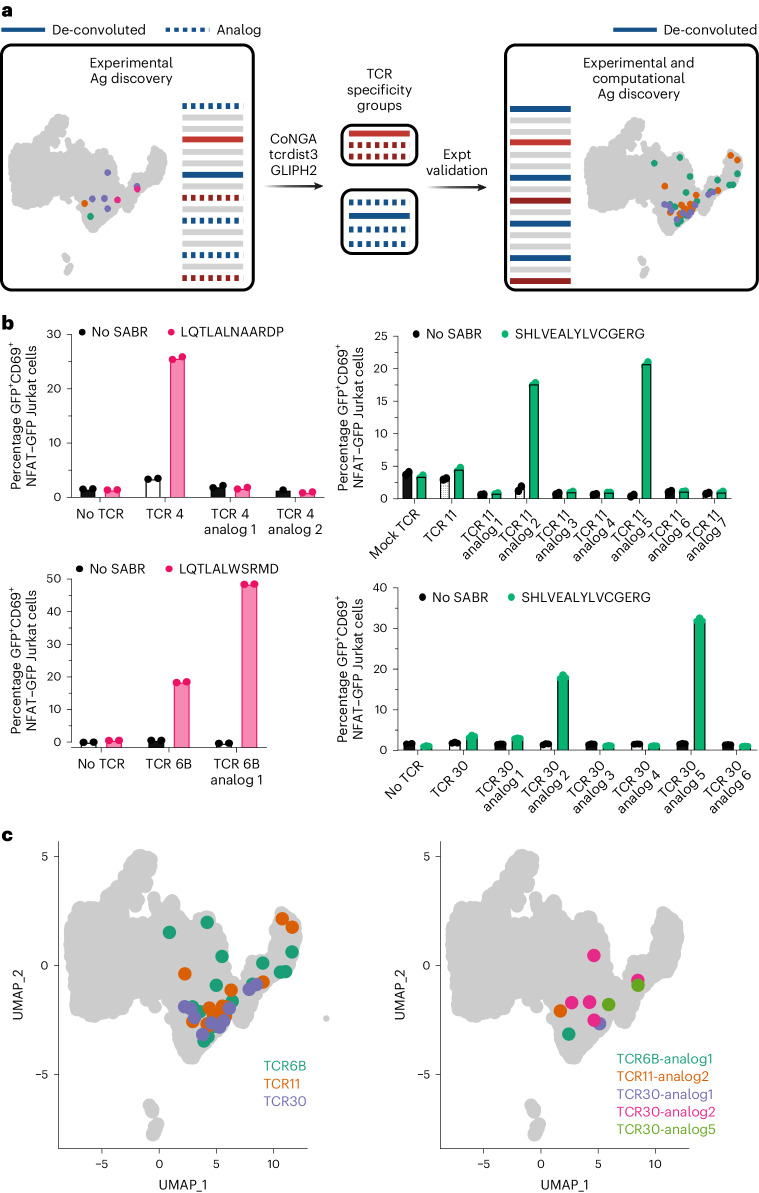
Computational prediction of antigen specificity amplifies SABR-II antigen discovery. **a**, Schematic of the implementation for computational prediction and validation of shared antigen specificity for TCRs from private datasets. Solid red and blue lines indicate experimentally de-convoluted TCRs, dashed blue and red lines indicate potential analogs that may share specificity with the experimental TCRs and gray lines indicate remaining TCRs. **b**, Single SABR assays for analogs of TCR4, TCR6B, TCR11 and TCR30 are shown. Bar plots depict mean of percentage GFP^+^CD69^+^ SABR-II NFAT–GFP Jurkat cells plotted from two technical replicates. **c**, Projection of antigen-specific TCRs onto the UMAP after validation of predicted antigen specificity (right) compared to original clones (left). Source data

**Extended Data Fig. 8:**
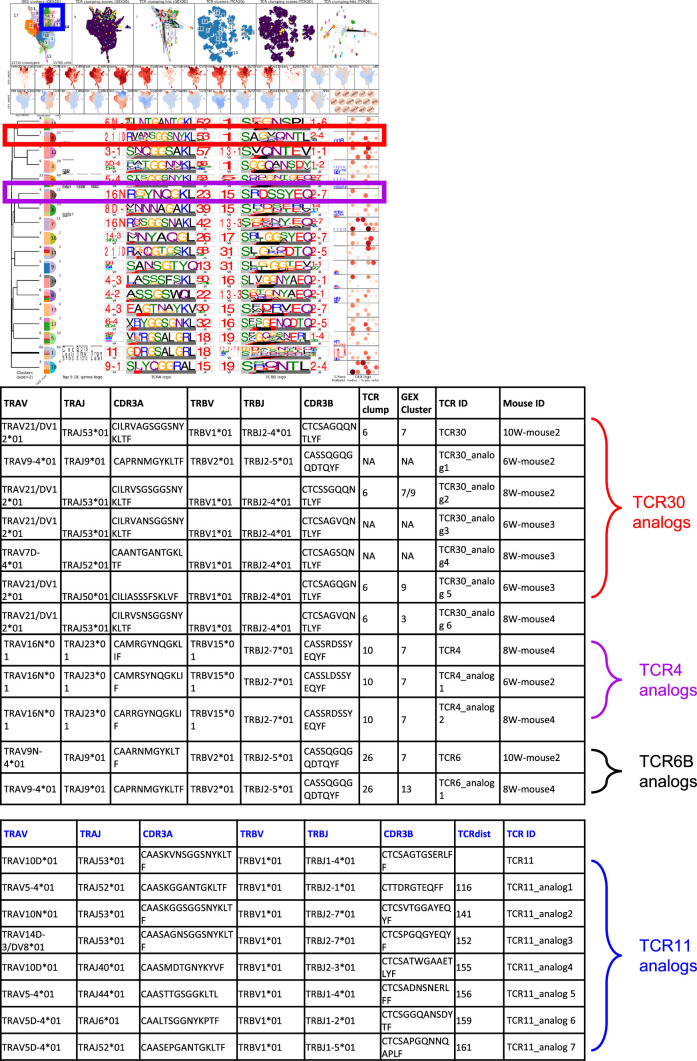
CoNGA, TCRdist results and identification of TCR analogs. The top three row of the CoNGA panel shows the GEX and TCR clusters with phenotype marker expression in each cluster. The TCR logo panel shows TCR clusters with logo representations of the TCRs that form these clusters. The purple, red, and blue rectangles indicate the TCR4 CoNGA cluster, TCR30 CoNGA cluster, and TCR11 gene expression clusters respectively. Each cluster’s TCRs which were selected for validation are listed in the tables below with the parental TCR. TCRs which lack GEX clusters in the TCR30 analog table were selected for validation by similarity determined through GLIPH2. TCR6B is not depicted in the figure because of the minimum clone size requirement for CoNGA, TCR11 mouse IDs are lost during CoNGA processing.

We hypothesized that computational grouping of TCR specificities may reveal closely related TCRs that potentially recognize the same epitope(s), similar to the reverse epitope discovery approach (Fig. 4a). In the absence of experimental antigen discovery, grouping of TCRs is not informative of reactivity; however, we hypothesized that TCRs that co-clustered with SABR-II de-convoluted TCRs bind to the same antigens. To test this, we used three TCR-similarity search algorithms: GLIPH2 (refs. ^38,69^), tcrdist3 (ref. ^37^) and CoNGA^39^. All three algorithms take slightly different approaches to group TCR sequences and generate clusters of TCR sequences that share high sequence similarity. In addition, CoNGA considers the transcriptional similarities among T cell clones. Using CoNGA, we defined TCR clusters for two TCRs, TCR4 and TCR6B, and identified analogs that slightly differed in sequences. Moreover, for TCR30, we were able to identify six TCR analogs that co-clustered in CoNGA analysis as well as GLIPH2. For TCR11, we first identified a gene expression (GEX) cluster that had ~50 TCRs that clustered based on gene expression. Using tcrdist3, we calculated the relative distance of each of these TCRs from TCR11 and selected the top seven clonotypes for expression. Together, 16 TCRs were identified as analogs of the experimentally de-convoluted TCRs (Extended Data Fig. 8). These TCRs were cloned and expressed in Jurkat cells. We performed co-incubation assays using single SABR-IIs and observed that 5 of 16 TCRs recognized the same epitopes as the parental TCRs (Fig. 4b). As a result, we were able to identify the cognate epitopes of five additional TCRs from our dataset that had otherwise not been selected for SABR-II screening based on our clonal expansion cutoff. Notably, the computationally identified and experimentally validated TCRs shared similar phenotypes as the experimentally de-convoluted TCRs (Fig. 4c). Therefore, we demonstrated that computational TCR similarity determinations could amplify experimental antigen discovery, leading to the deconvolution of 16 private TCRs de novo.

### Identifying new HIP epitopes using SABR-II libraries

**Fig. 5:**
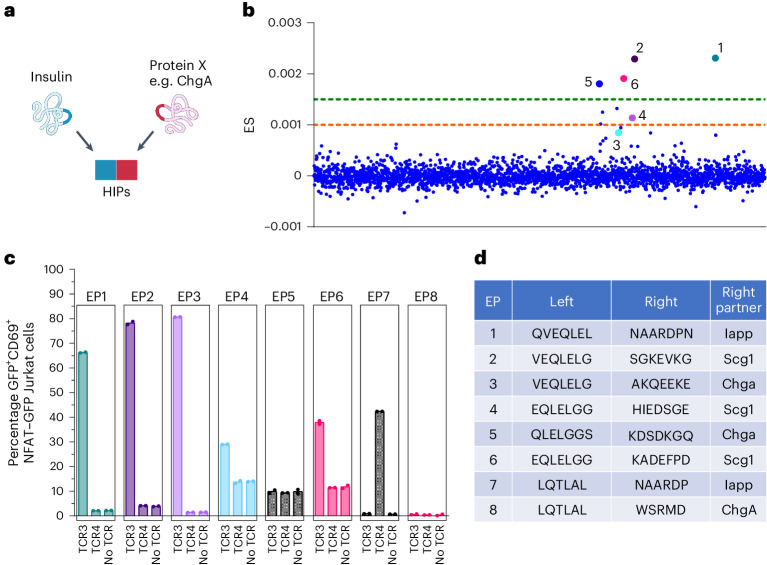
HIP target library for identification of TCR with new HIP specificity. **a**, Schematic of HIPs generated by the post-translational fusion between insulin and other secretory granule proteins. **b**, ES plot of TCR3 screen against the SABR-II HIP library. Six putative hits were selected (numbered and highlighted by the larger dots) for single SABR validation across the high (green dashed line) and low (orange dashed line) confidence zones. **c**, Single SABR assays of 14 amino acid HIPs spanning the fusion junctions incubated against TCR3 or TCR4 expressing Jurkat cells. Bar plots depict mean of percentage GFP^+^CD69^+^ SABR-II NFAT–GFP Jurkat cells plotted from two technical replicates (dots). **d**, The amino acid sequences of HIPs and their right-half source proteins for epitopes in **c** are indicated. Source data

Given the predominance of HIP-reactive TCRs, we hypothesized that there may be other TCRs that respond to HIPs that were not encoded in our initial library configuration. While the initial I-Ag7 SABR-II library consisted of a number of HIPs, HIP formation is thought to be more widespread in pancreatic β-cells^52,70^. Therefore, we sought to construct a defined, HIP-focused library to probe whether there were any undiscovered HIP-reactive TCRs that could be recognized by the clonally expanded TCR in our dataset. To test this, we utilized a published proteomic dataset, which predicted that several proteins that were highly expressed in secretory granules of β-cells may contribute to HIP formation^58^. Using their predictions, we built a theoretical HIP library, in which all possible ‘left’ halves of the insulin C chain derived from natural cleavage products were fused to ‘right’ halves derived from secretory granule proteins (Fig. 5a). This 2,561-epitope library (12–25 amino acids per epitope) consisted of only HIPs and a small number of positive control epitopes (Supplementary Table 5). We screened the top three clonally expanded TCRs (TCR1, TCR2 and TCR3) against this library, as these TCRs had not been de-convoluted using the original library. We did not observe any putative hits for TCR1 and TCR2; however, TCR3 yielded several high- and low-confidence hits, all of which have not previously been reported (Fig. 5b). To confirm that the HIP itself was important for the cognate interaction, we cloned single 14-mer epitopes into SABR-IIs consisting of seven amino acids of the left portion of the HIP and seven amino acids of the right portion of the HIP. In this way, no nine amino acids from either peptide sequence alone could occupy the binding pocket of I-Ag7, ensuring that TCR reactivity spanned the HIP junction^71^. Upon single SABR co-incubations, all but one of the tested hits for TCR3 showed reactivity (Fig. 5c). Of note, in all the epitopes that were tested, the ‘left’ half derived from insulin C was conserved, whereas the ‘right’ halves were derived from several other proteins (Fig. 5d). These results show that defined theoretical SABR-II libraries can be deployed for determining non-contiguous epitope reactivity as well as TCR promiscuity. Moreover, the promiscuous binding of TCR3 to HIPs corroborates evidence from other NOD mouse-derived TCRs reacting to multiple HIPs^72^.

### Technical advances afforded by SABR-II screens

**Extended Data Fig. 9:**
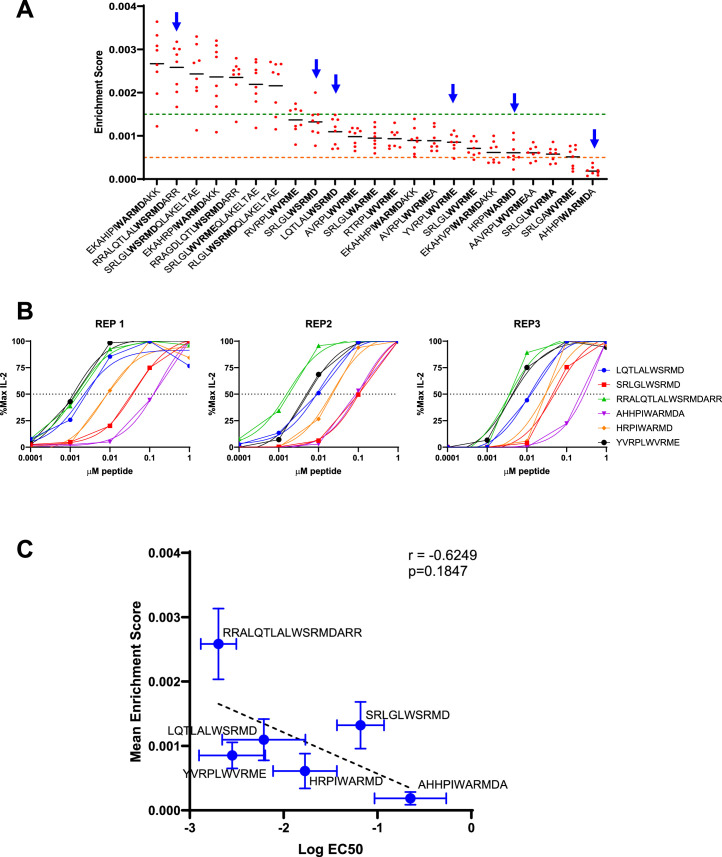
SABR-II screens are semi-quantitative readouts of functional avidity. A. Selected BDC2.5 epitopes for functional avidity measurements. As shown by blue arrows, 6 epitopes across range of ES were chosen from mean ES (bars) across 8 independent experiments (dots). B. Individual biological replicates of peptide pulsing experiments with BDC2.5 TCR-expressing 5KCs against selected peptides. The y-axis shows normalized murine IL-2 secretion across a range of peptide concentrations (x-axis). C. The mean (dots) and s.d. (error bars) of Log EC50 values (x-axis) from the 3 biological replicates in panel B plotted against the mean (dots) and s.d. of ES values across 8 biological replicates (y-axis). The r and p values for two-sided Pearson correlation (dashed line) are reported. Source data

**Extended Data Fig. 10:**
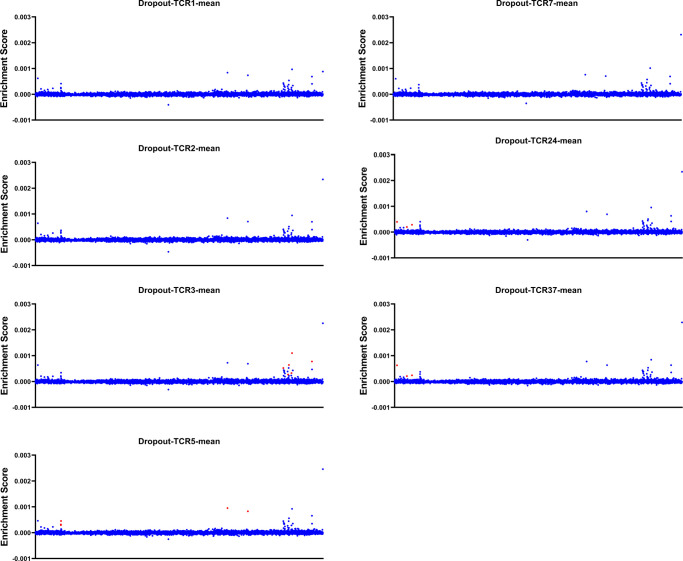
ES plots from TCR and library multiplexing screens. ES plots for each multiplexed sort where the dots represent the mean ES for each epitope in replicates (n = 6) where the target TCR was not included (dropout). The red dots indicate the cognate epitope of each TCR that were previously de-convoluted using individual TCR screens against the library. Source data

Finally, we sought to address two important aspects of antigen discovery techniques. First, we assessed whether SABR-II screens can directly read out the strength of TCR–pMHC binding. To that end, we selected six known BDC2.5 ligands across a range of ES values (Extended Data Fig. 9a) and we measured the functional avidity of their recognition by BDC2.5 TCR in vitro. Bone-marrow-derived dendritic cells were pulsed with a range of concentrations of peptides corresponding to the epitopes and used to present the peptides to BDC2.5 TCR-expressing 5KC cells. Secretion of mIL-2 was measured by ELISA and used to determine the functional avidity as EC_50_ (concentration of the peptide needed to induce half-maximal mIL-2) (Extended Data Fig. 9b). We observed that there was a modest negative correlation between the EC_50_ values of the epitopes and their ES values (Extended Data Fig. 9c). These results indicate that ES values can provide a semi-quantitative readout of the strength of interactions between TCRs and their cognate epitopes. Second, we evaluated whether we could increase the throughput of SABR-II screens by multiplexing TCRs and libraries at the cellular level. We combined the two previously described I-Ag7 libraries in equal proportions according to their size and used it as a single library. We also employed a dropout strategy, in which a mixture of seven TCRs was screened in replicate, where one TCR was left out in each replicate. After single enrichment, we determined the mean ES of all replicates that contained a given TCR and used it to identify the cognate epitope of that TCR (Extended Data Fig. 10 and Supplementary Table 6). Using this strategy, we were able to successfully recapitulate the results for four out of four TCRs previously identified in individual screens. The use of such a strategy will greatly enhance the throughput or SABR-II screens by reducing the hands-on sort time from 1 h per TCR to 20 min per TCR. These results show features that have been uniquely demonstrated by SABR-II screens and should increase the throughput of antigen discovery.

## Discussion

Here, we report SABR-IIs for CD4^+^ T cell antigen discovery, providing a robust method for screening a large number (1,000s to 10,000s) of epitopes. SABR-IIs can identify TCRs rapidly and can semi-quantitatively read out TCR–pMHC binding strengths. We have also shown that other non-T cell types can also be used to detect cognate interactions, expanding antigen discovery to professional APC-based platforms. Notably, SABR-II libraries can easily encode for deamidation and HIP formation, which are both post-translational modifications. Through this approach, we identified several new HIPs that were targeted by islet-infiltrating T cells and demonstrate an HIP-focused cell-based library strategy.

Moreover, we demonstrate a robust pipeline for reconstructing TCRs from scRNA-seq data and identifying their epitopes. The ability to start from and reconstitute TCRα/β sequences means that precious human samples are not wasted and can be assayed using additional methods. Furthermore, starting from scRNA-seq has the built-in advantage of leveraging the transcriptional information for each clone of an identified specificity, not limited by a few phenotypic surface markers or agnostic of the T cell’s function altogether. While we have chosen to profile the top expanded T cell clones in this study, we envision that future efforts can be focused on specific phenotypes of interest, such as regulatory T cells. In this way, both the environment from which the T cells are sampled and the properties of the T cells themselves will help further shape hypothesis-driven antigen discovery in autoimmune diseases such as T1D.

The ability to amplify antigen discovery using related TCRs by leveraging existing computational methods not only validates their utility but generates a positive-feedback loop for increased repertoire profiling and validation of TCR specificity. This will lead to an overall enlargement of the known epitope-specific TCR repertoire and provide incorporation of orthogonally obtained datasets for de novo antigen discovery. Finally, SABR-IIs in conjunction with SABRs, allow parallel antigen discovery for CD4^+^ and CD8^+^ T cells within the same platform and experiments.

We do wish to highlight the current limitations of our technique. The SABR-II in its current iteration is similar to the MCR-TCR platform^28,33^, which encodes for a signal emanating from MHC-II. There are several design differences that confer different capabilities to SABR-IIs, namely, the ability to perform single enrichments on larger libraries, the ability to multiplex TCRs and the ability to screen for both class I and II alleles. Notably, as the signaling domains of SABR-II are modular, SABR-IIs can be expressed and deployed in professional APCs; however, there are also key differences, such as lower library sizes, especially compared to the cDNA-generated libraries. As with the current cell-based epitope discovery methods, SABR-IIs cannot match the scale of yeast display, which can reach up to 10^8^ epitopes for profiling. Techniques such as TScan-II have shown genome-scale antigen discovery; however, they cannot be used for both class I and class II discovery in the same platform^35^. Therefore, while not required, certain a priori criteria such as MHC binding prediction, tissue expression patterns or known immunopeptidomic datasets greatly enhance SABR-II library design. SABR-II screens are currently performed as ‘few against many’ assays, allowing tens of TCRs to be screened in a single day. The computational prediction tools we used here also pose inherent limitations to our workflow. As shown, 10 of 16 computationally predicted TCRs did not recognize the same antigens as the parental TCRs. This may be due to the erroneous calling of clonotypes or due to the analog-binding variations of the epitopes tested here. Either way, while we were able to amplify experimental antigen discovery, caution must be taken to not presume that prediction equals actual binding.

While we showed de novo identification of the 11 top expanded TCRs out of 36, we did not identify the cognate epitopes of the remaining TCRs. This could be due to several reasons. First, we used a published MHC elution dataset, which inherently has high specificity but low sensitivity for detecting MHC-II-bound epitopes. Building new SABR-II libraries based on tissue-specific gene expression may benefit by casting a wider net in search of cognate epitopes. In addition, a hallmark of numerous autoreactive diseases is the reactivity to post-translationally modified epitopes^73,74^. While we were able to encode hybrid and deamidated epitopes in our SABR-II libraries, we are developing approaches to incorporate a wider range of chemical modifications. Finally, the antigen sensitivity of class I SABRs is inherently lower than those of TCRs. We expect that SABR-IIs may also have a similar limitation, where very-low-affinity antigens do not generate a strong SABR signal and remain below the limit of detection without further modification, such as the introduction of a disulfide trap to stabilize the MHC and fix weak binding registers in place.

In summary, this study demonstrates that wielding SABR-IIs for TCR-directed antigen discovery and amplifying discovery with existing computational methods is a powerful combination for understanding CD4^+^ T cell specificities. By increasing the ability to survey the T cell repertoire we envision a more comprehensive catalog of the T cell ‘reactome.’

## Methods

### Ethics statement

All animal work was performed as per Institutional Animal Care and Use Committee (IACUC) guidelines under an approved IACUC protocol (no. 20037102). All experimental work was performed according to the institutional biosafety committee protocols.

### Reagents and oligonucleotide primers

Reagents and oligonucleotide primers methods can be found in Supplementary Table 7. The lists of epitopes in the SABR-II libraries can be found in Supplementary Tables 3, 4 and 6.

### Cell lines and peptides

Jurkat cells (ATCC) and Daudi cells (ATCC) were cultured in R10 (RPMI 1640 medium (Corning) supplemented with 10% FBS (Gemini Bio) and 10 U ml^−1^ penicillin–streptomycin (Corning)). NFAT–GFP Jurkat cells were a kind gift from A. Weiss and Y. Chen and were cultured in R10 supplemented with 2 mg ml^−1^ Geneticin (Corning). HEK293T cells (ATCC) were cultured in D10 (DMEM (Corning) supplemented with 10% FBS (Gemini Bio) and 10 U ml^−1^ penicillin–streptomycin (Corning)). 5KC cells were a kind gift from M. Nakayama and were cultured in IMDM (Gibco) with 10% FBS (Gemini Bio) and penicillin–streptomycin. All cell culture was performed at 37 °C with 5% CO_2_ in a humid cell culture incubator. Primary CD4^+^ T cells were isolated from spleens for NOD mice using a STEMCELL murine CD4^+^ T cell-positive selection kit (STEMCELL Technologies) and cultured in R10 (RPMI 1640 medium (Corning) supplemented with 10% FBS (Gemini Bio) and 10 U ml^−1^ penicillin–streptomycin (Corning)) supplemented with 5 U ml^−1^ IL-2 (R&D Biosciences).

### Mice

Mice were housed in microisolator cages with up to five mice per cage in a 14-h light–10-h dark cycle. Temperatures of 65–75 °F (~18–23 °C) with 40–60% humidity were maintained. There was constant access to water. NOD/ShiLtJ (strain 001976, The Jackson Laboratory) mice were purchased at the age of 4 weeks. The mice were fed autoclaved rodent breeder diet (T. R. Last). Female mice were used for scRNA-seq and validation assays. For scRNA-seq, 6-, 8- or 10-week-old female mice were used. All animal work was performed under IACUC protocols in the Association for Assessment and Accreditation of Laboratory Animal Care-certified animal facility at the University of Pittsburgh.

### Construction of SABRs

SABRs were designed by assembling the individual component sequences in Snapgene (DNAstar). HLA allele chains were downloaded from IMGT and MHC allele chains were downloaded from UniprotKB. SignalP-5.0 (ref. ^75^) was used to predict the signal sequence and truncate it. The signaling domains were derived from the previously published SABR constructs^27^. Beta-chain-Signaling-2A-Alpha-chain fragments were assembled and codon-optimized using IDT’s codon optimization tool. BsmBI sites were replaced without affecting the amino acid sequences and EcoRI sites were added at the ends. A 2-kb stuffer fragment was also synthesized according to previously published sequences^27^. Open reading frames were synthesized as gBlocks (IDT) and assembled using PCR (KOD mastermix, Milipore Sigma) using the following primers: 2kb-Insert-gBlock-F; 2kb-Insert-gBlock-R; BsmBI-Insert-Fwd; and ClassII-Alpha-Rev. The assembled full-length inserts were gel purified (Takara), digested with EcoRI (NEB), ligated in EcoRI-digested pCCLc-MND-X (a kind gift from D.B. Kohn) and transformed using NEB-5α cells (NEB). Inserts were verified using MND_Input_Verify_F and MND_Input_Verify_R primers. Once full-length backbones were cloned, they were used to clone individual epitopes. To insert epitopes, SABR vectors were digested with BsmBI along with alkaline phosphatase (rSAP, NEB) to excise the 2-kb stuffer fragment. Two complementary oligonucleotides, SABR-epitope-F and SABR-epitope-R, were synthesized for each epitope. Oligonucleotides were annealed to each other, phosphorylated and ligated into the BsmBI-digested backbone (T4 Ligase, NEB) and transformed in NEB-5α cells (NEB). For cloning SABR libraries, oligonucleotide pools containing overhangs (oligonucleotide epitope primer) were synthesized via Twist Biosciences. The pool was amplified using ClassII-Oligo-Fwd and ClassII-Oligo-Rev and cloned in a BsmBI-digested backbone using Infusion HD cloning (Takara). Bacteria were plated on LB agar containing 100 μg ml^−1^ carbenicillin (Life Technologies), grown overnight and single colonies were selected for verification by Sanger sequencing (Azenta). Successful clones were used to inoculate liquid culture for overnight growth followed by plasmid minipreps (Zyppy miniprep kit, Zymo). Pooled libraries were subjected to maxipreps (Nucleobond Maxiprep EF kit, Takara). Library coverage was determined by comparing the number of total colonies transformed to the number of epitopes encoded in the library. For B cell receptor SABRs, the protein sequences CD79A and CD79B domains were obtained from UniprotKB and fused with full-length MHC-II chains and obtained via commercial synthesis (Twist Biosciences). Epitopes were cloned in the B cell receptor SABR backbone as described above, except that the stuffer fragment was removed using XhoI digestion (NEB).

### scRNA-seq of islet-infiltrating T cells and analysis

NOD mice were killed by CO_2_ asphyxiation and immediately dissected for pancreas perfusion and individual islet picking as previously decsribed^66^. Pancreas perfusion was performed under a dissecting microscope. The pancreatic duct was clamped using surgical clamps and 3 ml 600 U ml^−1^ Collagenase IV (Gibco) dissolved in HBSS (Gibco) was injected using a 30G needle. Perfused pancreata were collected and incubated at 37 °C for 30 min. After the incubation, HBSS with R10 was added to quench collagenase. After washing twice with HBSS + R10, the tissue was plated on a 10-cm plate and individual islets were picked using a micropipette. Islets were then incubated in dissociation buffer (Gibco), centrifuged and resuspended in the staining mix (1:500 dilution of anti-Thy1.2-BV605 + 1:500 dilution of Live/Dead-APC-Cy7 and 1:100 dilution of cell-hashing TotalSeq antibodies (BioLegend)). After staining, the cells were resuspended in PBS + 0.04% BSA (Millipore Sigma) and sorted on a BD FACS Aria III sorter. After sorting the cells, they were counted and processed for scRNA-seq. Cells were processed using 10× 5′ single-cell gene expression kit v3 in a Chromium controller according to the manufacturer’s protocols. V(D)J enrichment was performed using the single-cell 5′ VDJ enrichment kit according to the manufacturer’s protocols. Libraries were sequenced on a HiSeq4000 (Novogene) with a 70:20:10 mix for gene expression:VDJ:hashing libraries. Sequence data were downloaded on the Joglekar laboratory server and aligned to the mouse genome (Mm10) using CellRanger v.4.0.0 (10x Genomics). TCR annotation was performed using CellRanger vdj using mouse GRCm38 assembly. All three time points were sequenced and processed separately. CellRanger and CellRanger vdj output files were used as inputs in Seurat^62^ for normalization, scaling and dimensionality reduction. The packaged scRepertoire was used for TCR clonotype calling and analyses. The data were normalized using NormalizeData and scaled using ScaleData functions in Seurat. The scRepertoire^63^ functions combineTCR and combineExpression were used to add TCR clonotypes to each cell. The HTODemux function in Seurat was used to demultiplex cell hashes and assign the correct mouse identity to each cell. At this point, all three time points were merged in Seurat using the merge function. After merging, integration was performed using FindIntegrationAnchors and IntegrateData functions. Principal-component analysis was performed using RunPCA. The top 20 principal components were used for UMAP, followed by cluster identification using FindNeighbors and FindClusters. CD4^+^ T cells were subsetted using FeatureScatter and CellSelector functions and reclustered. Cluster markers were defined by the FindAllMarkers function. Clonotype data were sorted according to expansion and exported as a csv file. UMAP representations with clonotypes were generated using the highlightClonotypes function in scRepertoire. Differentially expressed genes were identified using the FindMarkers function using DESeq2 statistics and represented using EnhancedVolcano function. For the related manuscript^67^ (Xiao, Rohimikollu and Rosengart et al.), single (1), low (2–9) and medium (≥10) clonotypes were subsetted in Seurat and exported as Seurat objects for further analyses. All scRNA-seq analyses were performed using RStudio (v.2023.12.1+402).

### TCR reconstruction and synthesis

TCR Vα, Jα, Vβ and Jβ alleles along with CDR3α and CDR3β sequences were used as the input to reconstruct full-length TCR sequences using the TCRgen_mouse.opt_v2.py script (available on GitHub at https://github.com/joglekar-lab/SABR-II). Mouse reference sequences were downloaded from IMGT. Full-length TCR sequences (TCRα-2A-TCRβ) flanked by EcoRI site and truncated at the BlpI site in Cb were synthesized as gene fragments via Twist Biosciences. TCR gene fragments were amplified using TCR-gene-fwd and TCR-gene-rev primers and subcloned using a pMIG-II vector containing BDC2.5 TCR (Vignali laboratory) using EcoRI-BlpI. Successful cloning was verified using Sanger sequencing (Azenta).

### TCR similarity determinations

Exported clonotypes were used as inputs for GLIPH2 (ref. ^69^). For CoNGA, the merged dataset was exported as a .h5ad file and used as an input along with the CellRanger vdj output file. CoNGA analysis was performed using default parameters^39^. Pairwise relative distances among TCRs were calculated using tcrdist3 (ref. ^37^). CoNGA, tcrdist3 and GLIPH2 output files were searched manually for analogs that co-cluster with experimentally de-convoluted TCRs. Analogs were synthesized and cloned as described above.

### Generation and cloning of SABR libraries

To generate the I-Ag7 restricted SABR library, we combined all Immune Epitope Database epitopes with a published immunopeptidome generated by Wan et al.^51^. Sequences were filtered remove all post-translational modifications except deamidation and HIPs and trimmed between 9–25 amino acid lengths. For the insulin C HIP and HLA-DQ8 library, non-contiguous epitopes from Wiles et al.^66^ as well as all Immune Epitope Database epitopes were combined to generate the epitope list. Epitope sequences were back-translated using the backtranslate_fast.py script.

### Lentiviral vector production and transduction

Lentiviral vectors to express SABRs or TCRs were packaged via previously described procedures^27,76^. In brief, HEK293T cells were plated in six-well plates at 1 × 106 cells per well. After 24 h, cells were transfected with a mixture of the lentiviral shuttle plasmid (1 μg per well), pMDG-VSVG (0.2 μg per well) and pCMV-RD8.9 (1 μg per well) (both kind gifts from D.B. Kohn) using TransIT-293 (Mirus Bio) and OPTI-MEM (Life Technologies) using the TransIT-293 manufacturer’s protocol. After 3 days, viral supernatant was collected and filtered through 0.45-μm syringe filters (Millipore). When possible, the freshly filtered virus was used to transduce 1 × 10^6^ Jurkat cells per ml of the virus. Occasionally, the virus was stored at −80 °C until use. For NFAT–GFP Jurkat cells, Geneticin was added 24 h following transduction.

### Retroviral vector production and transduction

Retroviral vectors (pMIG-II) to express TCRs were packaged via previously described procedures^77^. In brief, HEK293T cells were plated in six-well plates at 1 × 10^6^ cells per well. After 24 h, the cells were transfected with a mixture of the retroviral shuttle (1 μg per well), pRD114 (0.8 μg per well) and pHIT60 (1 μg per well) using TransIT-293 (Mirus Bio) and OPTI-MEM (Life Technologies). The following day, viral supernatant was collected and filtered through 0.45-μm syringe filters (Millipore). Transduction of 2.5 × 10^5^ Jurkat cells was performed using RetroNectin (Takara) binding according to the manufacturer’s protocol using the filtered virus. For primary murine CD4^+^ T cells and 5KC cells, Phoenix-ECO cells (ATCC) were plated in six-well plates at 1 × 10^6^ cells per well. After 24 h, the cells were transfected with the retroviral shuttle (2.5 μg per well) using TransIT-293 (Mirus Bio) and OPTI-MEM (Life Technologies) using the TransIT-293 manufacturer’s protocol. At 48 h after transfection viral supernatant was collected and filtered through 0.45-μm syringe filters (Millipore). Transduction of 2.5 × 10^5^ 5KC or primary murine CD4^+^ T cells was performed using RetroNectin (Takara) binding according to the manufacturer’s protocol using the filtered virus. Before transduction, primary murine CD4^+^ T cells were stimulated and grown for 24 h on 24-well plates coated with RetroNectin, 2 μg ml^−1^ anti-CD3ε (BioLegend) and 1 μg ml^−1^ anti-CD28 (BioLegend).

### Co-culture assays

For SABR library screens, 3 × 10^6^ NFAT–GFP Jurkat cells expressing the SABR library were labeled with CellTrace Violet (BioLegend) according to the manufacturer’s protocol before incubation with 3 × 10^6^ Jurkat cells expressing the TCR of interest. These mixtures were incubated in a six-well plate for 16–20 h. Cells were stained with anti-CD69-APC-Cy7 where indicated (BioLegend) and the top 1–2% of GFP^+^CD69^+^ cells were sorted for genomic DNA extraction, indexing and sequencing (see below). Multiplexed assays were scaled on a per-TCR basis (for example 3 × 10^6^ for each of three TCRs against 9 × 10^6^ library cells). For single SABR assays, unless otherwise defined, 5 × 10^5^ SABR expressing NFAT–GFP Jurkat cells (or 5KC cells) were labeled with CellTrace Violet (BioLegend) according to the manufacturer’s protocol before incubation with 5 × 10^5^ TCR-expressing Jurkat cells (or 5KC cells) in a round-bottom 96-well plate for 16–20 h. Cells were stained with anti-CD69-APC-Cy7 when indicated and acquired on the Attune NxT flow cytometer (Thermo Fisher Scientific). All flow analysis was performed using FlowJo (BD). For Daudi cell co-culture, 1 × 10^6^ Jurkat cells expressing the TCR of interest were incubated with 1 × 10^6^ Daudi cells expressing a SABR of interest for 3 days. On day 3, cells were labeled with anti-RT1B-PE and anti-Fas-APC-Cy7 before being acquired on the Attune NxT flow cytometer.

### High-throughput sequencing and analysis

Genomic DNA was extracted from sorted cells immediately after sorting, using the PureLink genomic DNA extraction kit (Life Technologies). The integrated SABR vectors were amplified with KOD polymerase (Millipore) and two rounds of amplification. In the first round, IDT-UD-SABR-C2-F and IDT-UD-SABR-C2-R primers were used to amplify the epitope. In the second round, UDI0001-R and UDI0001-F primers (representative of index 1) were used to add Illumina unique dual indexes (UDIs) to the amplicons. A different UDI was used for each sample. The reactions were pooled and purified with the NucleoSpin gel and PCR purification kit (Takara). The purified PCR product was checked before sequencing using 2% agarose gel and subjected to sequencing on a HiSeq4000 (Fulgent Genetics). Unaligned reads generated by the sequencer were stored in FASTQ files. FASTQ files were concatenated to generate one file for read1 and read2 each. The sequences were demultiplexed into individual indexes using demultiplex_dual.py. Epitopes were extracted and scored using epitope_extract_fastq_v1.1.py and merge_counts_split_v2.1.py. The ES was calculated using Microsoft Excel (Microsoft) workbooks.

### Peptide pulsing assays

Bone-marrow-derived dendritic cells (BMDCs) were generated according to Abcam’s protocol (https://www.abcam.com/protocols/bmdc-isolation-protocol) by isolating bone marrow from NOD mice and differentiating these cells in granulocyte–macrophage colony-stimulating factor (R&D Systems) for 7 days. On day 7, 2 × 10^4^ BMDCs were resuspended in R10 and plated in a flat-bottom 96-well plate. Tenfold serial dilutions of each peptide were added to the BMDCs and left to incubate for 1 h. After 1 h, 5 × 10^4^ 5KC or primary murine CD4^+^ T cells were added to the peptide-pulsed BMDCs. The assay was left to incubate for 24 h, at which point cells were spun down, supernatant was collected and used for mIL-2 detection with the LEGEND MAX Mouse IL-2 ELISA kit (BioLegend). Peptides were custom ordered from GenScript.

### Statistical analysis

Flow cytometry plots were analyzed with FlowJo v.10. Statistical analyses and graphical representations were generated by Microsoft Excel and GraphPad Prism v.9 and v.10 (GraphPad).

### Reporting summary

Further information on research design is available in the Nature Portfolio Reporting Summary linked to this article.

## Online content

Any methods, additional references, Nature Portfolio reporting summaries, source data, extended data, supplementary information, acknowledgements, peer review information; details of author contributions and competing interests; and statements of data and code availability are available at 10.1038/s41592-024-02255-0.

### Supplementary information

Reporting Summary

Supplementary Table 1 Lists of epitopes for I-Ag7 SABR-II library.

Supplementary Table 2 Lists of epitopes for DQ8 SABR-II library and ES.

Supplementary Table 3 TCR alleles and CDR3 sequences for top clonally expanded TCRs.

Supplementary Table 4 ES calculations for all TCRs.

Supplementary Table 5 Lists of epitopes for I-Ag7 HIP library and ES.

Supplementary Table 6 ES from dropout analysis.

Supplementary Table 7 Reagents used in the study.

### Source data

Source Data Fig. 1 Statistical source data.

Source Data Fig. 2 Statistical source data.

Source Data Fig. 3 Statistical source data.

Source Data Fig. 4 Statistical source data.

Source Data Fig. 5 Statistical source data.

Source Data Extended Data Fig. 1 Statistical source data.

Source Data Extended Data Fig. 2 Statistical source data.

Source Data Extended Data Fig. 3 Statistical source data.

Source Data Extended Data Fig. 4 Statistical source data.

Source Data Extended Data Fig. 5 Statistical source data.

Source Data Extended Data Fig. 6 Statistical source data.

Source Data Extended Data Fig. 7 Statistical source data.

Source Data Extended Data Fig. 9 Statistical source data.

Source Data Extended Data Fig. 10 Statistical source data.

## Data Availability

Sequencing data are available on the Gene Expression Omnibus under accession ID GSE247410. SABR-II plasmids, SABR-II libraries and TCRs will be made available upon request, given the large number of them. The individual sequences of epitopes as well as sufficient information to reconstruct the TCRs are provided in supplementary files. Source data are provided with this paper.

## Code availability

All necessary scripts are deposited to *GitHub* at https://github.com/joglekar-lab/SABR-II.
