# Supplementary material for: De novo identification of CD4+ T cell epitopes
Source: Nat Methods. 2024 Apr 24;21(5):846–56. doi: 10.1038/s41592-024-02255-0 (PMC11093748; doi:10.1038/s41592-024-02255-0)
Supplement: Supplementary file 1 — Reporting Summary [file 41592_2024_2255_MOESM1_ESM.pdf]

nature portfolio

Corresponding author(s): Joglekar, Alok V.

Last updated by author(s): Feb 28, 2024

Reporting Summary

Nature Portfolio wishes to improve the reproducibility of the work that we publish. This form provides structure for consistency and transparency in reporting. For further information on Nature Portfolio policies, see our Editorial Policies and the Editorial Policy Checklist.

Statistics

For all statistical analyses, confirm that the following items are present in the figure legend, table legend, main text, or Methods section.

n/a	Confirmed	
☐	☒	The exact sample size (*n*) for each experimental group/condition, given as a discrete number and unit of measurement
☐	☒	A statement on whether measurements were taken from distinct samples or whether the same sample was measured repeatedly
☐	☒	The statistical test(s) used AND whether they are one- or two-sided<br>*Only common tests should be described solely by name; describe more complex techniques in the Methods section.*
☒	☐	A description of all covariates tested
☒	☐	A description of any assumptions or corrections, such as tests of normality and adjustment for multiple comparisons
☐	☒	A full description of the statistical parameters including central tendency (e.g. means) or other basic estimates (e.g. regression coefficient) AND variation (e.g. standard deviation) or associated estimates of uncertainty (e.g. confidence intervals)
☐	☒	For null hypothesis testing, the test statistic (e.g. *F*, *t*, *r*) with confidence intervals, effect sizes, degrees of freedom and *P* value noted<br>*Give P values as exact values whenever suitable.*
☒	☐	For Bayesian analysis, information on the choice of priors and Markov chain Monte Carlo settings
☒	☐	For hierarchical and complex designs, identification of the appropriate level for tests and full reporting of outcomes
☐	☒	Estimates of effect sizes (e.g. Cohen's *d*, Pearson's *r*), indicating how they were calculated

Our web collection on statistics for biologists contains articles on many of the points above.

Software and code

Policy information about availability of computer code

Data collection	no software used for data collection
Data analysis	Cellranger-4.0.0 was used for alignment of sequence reads<br>Seurat v4.2.0 and scRepertoire v1.7.2 was used for single cell RNA seq data analysis<br>TCR reconstruction was done using a custom script: TCRgen_mouse.opt_v2.py and imgt_tcr_mouse.nuc.fa<br>SABR library design was done using a custom script: backtranslate_fast_noU_upto25.py<br>SABR screen data were analyzed using demultiplex_dual.py; epitope_extract_fastq_v1.1.py; merge_counts_split_v2.1.py<br>All the custom scripts and an R-studio (version 2023.12.1+402) notebook describing the scRNAseq analysis are deposited on Github: https://github.com/joglekar-lab/SABR-II<br>The code availability statement is included in the manuscript.<br><br>Graphpad Prism v9 and v10 were used for data analysis.<br><br>CoNGA, tcrdist3, GLIPH2 were used as per the published instructions.

For manuscripts utilizing custom algorithms or software that are central to the research but not yet described in published literature, software must be made available to editors and reviewers. We strongly encourage code deposition in a community repository (e.g. GitHub). See the Nature Portfolio guidelines for submitting code & software for further information.

Data

Policy information about availability of data

All manuscripts must include a data availability statement. This statement should provide the following information, where applicable:

- Accession codes, unique identifiers, or web links for publicly available datasets
- A description of any restrictions on data availability
- For clinical datasets or third party data, please ensure that the statement adheres to our policy

Single cell RNA sequencing data are publicly available on Gene Expression Omnibus (Accession: GSE247410)
Mouse genome GRCm39 was used for alignment of scRNAseq data (https://www.ncbi.nlm.nih.gov/datasets/genome/GCF_000001635.20/)
TCR sequences and epitope sequences are available as supplmentary data files and their details are included in the manuscript at the appropriate places.

Human research participants

Policy information about studies involving human research participants and Sex and Gender in Research.

Reporting on sex and gender	N/A
Population characteristics	N/A
Recruitment	N/A
Ethics oversight	N/A

Note that full information on the approval of the study protocol must also be provided in the manuscript.

Field-specific reporting

Please select the one below that is the best fit for your research. If you are not sure, read the appropriate sections before making your selection.

☒ Life sciences ☐ Behavioural & social sciences ☐ Ecological, evolutionary & environmental sciences

For a reference copy of the document with all sections, see nature.com/documents/nr-reporting-summary-flat.pdf

Life sciences study design

All studies must disclose on these points even when the disclosure is negative.

Sample size	A sample size of 40 TCRs was chosen arbitrarily based on the expansion size
Data exclusions	None
Replication	3 biological replicates were done for validation assays<br>3 technical replicates were done for each SABR-II screen and averaged for a given TCR<br>All attempts at replication were successful.
Randomization	Randomization was not relevant to the study, as each TCR was screened independently of others.
Blinding	Blinding was not performed, because the specificity of a given TCR is not known a priori. As each TCR is screened independently of other TCRs, no bliding was required.

Reporting for specific materials, systems and methods

We require information from authors about some types of materials, experimental systems and methods used in many studies. Here, indicate whether each material, system or method listed is relevant to your study. If you are not sure if a list item applies to your research, read the appropriate section before selecting a response.

Materials & experimental systems

n/a	Involved in the study
☐	☒ Antibodies
☐	☒ Eukaryotic cell lines
☒	☐ Palaeontology and archaeology
☐	☒ Animals and other organisms
☒	☐ Clinical data
☒	☐ Dual use research of concern

Methods

n/a	Involved in the study
☒	☐ ChIP-seq
☐	☒ Flow cytometry
☒	☐ MRI-based neuroimaging

Antibodies

Antibodies used

Anti-mouse TCRbeta PE, Clone H57-597; 109208 (Biolegend)
Anti-rat RT1B, PE, Clone OX-6; 205308 (Biolegend)
Anti-mouse I-A/I-E APC-Cy7, Clone M5/114.15.2; 107628 (Biolegend)
Anti-human CD69 APC-Cy7 (Clone FN50) :310914 (Biolegend)
Anti-mouse CD25, APC-Cy7, Clone PC61: 102025 (Biolegend)
Anti mouse Thy1.2, BV605; 105343 (Biolegend)
TotalSeq™-C0301 anti-mouse Hashtag Antibody,Clone M1/42; 30-F11; Tag # 1-10 ;155861-155879 (Biolegend)
Anti-human Fas, APC-Cy7 (Clone DX2); 305635 (Biolegend)
Ultra-LEAF Anti-mouse CD3 (Clone 145-2C11); 100340 (Biolegend)
Ultra-LEAF Anti-mouse CD28 (Clone 37.51); 102116 (Biolegend)

Validation

All antibodies were obtained from commercial vendors and were validated by the vendors prior to us purchasing them.
Anti-mouse TCRbeta PE, Clone H57-597; 109208 (Biolegend)
https://d1spbj2x7qk4bg.cloudfront.net/Default.aspx?ID=13064&pdf=true&displayInline=true&ProductID=272&leftRightMargin=15&topBottomMargin=15&filename=PE%20anti-mouse%20TCR%20%CE%B2%20chain%20Antibody.pdf&v=20240208073156

Anti-rat RT1B, PE, Clone OX-6; 205308 (Biolegend)
https://d1spbj2x7qk4bg.cloudfront.net/Default.aspx?ID=13064&pdf=true&displayInline=true&ProductID=5725&leftRightMargin=15&topBottomMargin=15&filename=PE%20anti-rat%20RT1B%20Antibody.pdf&v=20231114073227

Anti-mouse I-A/I-E APC-Cy7, Clone M5/114.15.2; 107628 (Biolegend)
https://d1spbj2x7qk4bg.cloudfront.net/Default.aspx?ID=13064&pdf=true&displayInline=true&ProductID=5966&leftRightMargin=15&topBottomMargin=15&filename=APC/Cyanine7%20anti-mouse%20I-A/I-E%20Antibody.pdf&v=20240207103033

Anti-human CD69 APC-Cy7 (Clone FN50) :310914 (Biolegend)
https://d1spbj2x7qk4bg.cloudfront.net/Default.aspx?ID=13064&pdf=true&displayInline=true&ProductID=1917&leftRightMargin=15&topBottomMargin=15&filename=APC/Cyanine7%20anti-human%20CD69%20Antibody.pdf&v=20240207043300

Anti-mouse CD25, APC-Cy7, Clone PC61: 102025 (Biolegend)
https://d1spbj2x7qk4bg.cloudfront.net/Default.aspx?ID=13064&pdf=true&displayInline=true&ProductID=3902&leftRightMargin=15&topBottomMargin=15&filename=APC/Cyanine7%20anti-mouse%20CD25%20Antibody.pdf&v=20240207103033

Anti mouse Thy1.2, BV605; 105343 (Biolegend)
https://d1spbj2x7qk4bg.cloudfront.net/Default.aspx?ID=13064&pdf=true&displayInline=true&ProductID=13864&leftRightMargin=15&topBottomMargin=15&filename=Brilliant%20Violet%20605%E2%84%A2%20anti-mouse%20CD90.2%20(Thy1.2)%20Antibody.pdf&v=20240106073142

TotalSeq™-C0301 anti-mouse Hashtag Antibody,Clone M1/42; 30-F11; Tag # 1-10 ;155861-155879 (Biolegend)
https://d1spbj2x7qk4bg.cloudfront.net/Default.aspx?ID=13064&pdf=true&displayInline=true&ProductID=18447&leftRightMargin=15&topBottomMargin=15&filename=TotalSeq%E2%84%A2-C0310%20anti-mouse%20Hashtag%2010%20Antibody.pdf&v=20240208073156

Anti-human Fas, APC-Cy7 (Clone DX2); 305635 (Biolegend)
https://d1spbj2x7qk4bg.cloudfront.net/Default.aspx?ID=13064&pdf=true&displayInline=true&ProductID=12141&leftRightMargin=15&topBottomMargin=15&filename=APC/Cyanine7%20anti-human%20CD95%20(Fas)%20Antibody.pdf&v=20240208073156

Ultra-LEAF Anti-mouse CD3 (Clone 145-2C11); 100340 (Biolegend)
https://d1spbj2x7qk4bg.cloudfront.net/Default.aspx?ID=13064&pdf=true&displayInline=true&ProductID=7722&leftRightMargin=15&topBottomMargin=15&filename=Ultra-LEAF%E2%84%A2%20Purified%20anti-mouse%20CD3%CE%B5%20Antibody.pdf&v=20240208073156

Ultra-LEAF Anti-mouse CD28 (Clone 37.51); 102116 (Biolegend)

https://d1spbj2x7qk4bg.cloudfront.net/Default.aspx?ID=13064&pdf=true&displayInline=true&ProductID=7733&leftRightMargin=15&topBottomMargin=15&filename=Ultra-LEAF%E2%84%A2%20Purified%20anti-mouse%20CD28%20Antibody.pdf&v=20240124061504

Eukaryotic cell lines

Policy information about cell lines and Sex and Gender in Research

Cell line source(s)	Phoenix Eco Cells; CRL-3214; ATCC<br>HEK-293T CRL-3216; ATCC<br>Jurkat cells TIB-152; ATCC<br>Daudi cells CCL-213; ATCC<br>5KC cells: Nakayama Lab, Barbara Davis Center for Diabetes, University of Colorado Anschutz Medical Campus<br>NFAT-GFP-Jurkat cells: Weiss Lab, University of California, San Francisco
Authentication	None
Mycoplasma contamination	Mycoplamsa negative by Hoescht Staining
Commonly misidentified lines (See ICLAC register)	None

Animals and other research organisms

Policy information about studies involving animals; ARRIVE guidelines recommended for reporting animal research, and Sex and Gender in Research

Laboratory animals	NOD/ShiLtJ; 001976; The Jackson Laboratory<br>Age 4-10 weeks, Sex: female<br>Mice were housed in microisolator cages with upto 5 mice per cage; 14-hour light/10-hour dark cycle was used. Temperatures of 65-75°F (~18-23°C) with 40-60% humidity were maintained. There was constant access to water.
Wild animals	No wild animals were used in this study
Reporting on sex	Female NOD mice are much more prone to T1D, therefore female mice were used for single cell RNA sequencing.
Field-collected samples	No field collected samples were not used in this study
Ethics oversight	Animal research was approved by the IACUC at University of Pittsburgh. The full information on the protocol is added to the manuscript

Note that full information on the approval of the study protocol must also be provided in the manuscript.

Flow Cytometry

Plots

Confirm that:

☒ The axis labels state the marker and fluorochrome used (e.g. CD4-FITC).

☒ The axis scales are clearly visible. Include numbers along axes only for bottom left plot of group (a 'group' is an analysis of identical markers).

☒ All plots are contour plots with outliers or pseudocolor plots.

☒ A numerical value for number of cells or percentage (with statistics) is provided.

Methodology

Sample preparation	Cells were harvested by centrifugation, resuspended in PBS + 2% FBS and stained with antibody mixes for 20 min at 4 deg celcius. After 20 min, cells were washed 2x with PBS + 2% FBS and filtered before flow cytometry
Instrument	Attune NxT with CytKickMax was used for analytical flow cytometry. BD Aria was used for sorting cells
Software	Manufacturer's default software was used, Analysis was done in Flowjo v10
Cell population abundance	Individual experiments ranged in their purity as indicated in supplementary figures.
Gating strategy	The gating strategy for analytical flow cytometry: Gate on FSC/SSC for lymphocytes; SSC-A vs SSC-H for singlets, and individual stains were gated based on negative controls

☒ Tick this box to confirm that a figure exemplifying the gating strategy is provided in the Supplementary Information.
